# Reduced version of the MBGR protocol: validity based on content and the response processes

**DOI:** 10.1590/2317-1782/e20250355en

**Published:** 2026-07-31

**Authors:** Asenate Soares de Matos Pereira, Herick Santos Assis, Paola Pereira Leite, Katia Flores Genaro, Giédre Berretin-Felix

**Affiliations:** 1 Departamento de Fonoaudiologia, Faculdade de Odontologia de Bauru – FOB, Universidade de São Paulo – USP - Bauru (SP), Brasil.

**Keywords:** Speech-Language Pathology, Clinical Protocols, Validation Study, Health Evaluation, Stomatognathic System

## Abstract

**Purpose:**

Considering the clinical need for greater assessment efficiency, this study aimed to present the short version of the MBGR Protocol, addressing the stages of content-based validity evidence and response process validation.

**Methods:**

A preliminary version of the reduced MBGR Protocol was developed based on the original version and subjected to the Delphi methodology. Content validation was conducted in two rounds, using a Likert scale to assess the relevance and representativeness of the items. The Content Validity Index (CVI) was calculated. For the response process validation, 11 speech- language pathologists (SLPs) were trained, and the protocol was applied to 11 patients, with video recording. Subsequently, the professionals completed a questionnaire, and the responses were analyzed and discussed with an expert committee. Statistical analysis was performed to compare the reduced and original versions, using paired t-tests or the Wilcoxon test, with a significance level set at p<0.05.

**Results:**

The initial proposal of the reduced protocol included 36 items. Of the 11 invited experts, seven analyzed and provided feedback on each item, leading to the exclusion of 17 items. The content validity evidence yielded a global CVI of 87%. During the response process evaluation, the SLPs reported adequate understanding and applicability of the short version. Most professionals considered the original protocol too extensive and the reduced version more feasible for clinical practice. The suggestions were mostly incorporated by the authors, resulting in adjustments and exclusions that finalized the reduced protocol, with a total score ranging from 0 to 137 points.

**Conclusion:**

The reduced version of the MBGR Protocol demonstrated evidence of content validity and response process validation, resulting in a final version with a shorter application time, making it more suitable for clinical use.

## INTRODUCTION

The Orofacial Myofunctional Therapy (OMT) area focuses on diagnosing orofacial myofunctional disorders through functional and morphological examinations of the stomatognathic system (SS)^(1)^. To make this practice less prone to errors^(1)^, the use of validated protocols to guide clinical observation and monitor the evolution of the patient throughout the treatment process is fundamental^(2)^.

Among the validated and existing instruments in OMT, the MBGR Orofacial Myofunctional Evaluation Protocol^(3)^ includes, in its proposal, the general aspects of the SS and the orofacial functions of breathing, chewing, swallowing, and speech. This protocol consists of two main parts: Clinical History and Orofacial Myofunctional Examination, encompassing the evaluation of morphofunctional aspects and functions. In addition, it includes documentation, involving photographs and videos, for recording and later analysis.

The MBGR Protocol^(3)^ has been widely used for the assessment and diagnosis of orofacial myofunctional disorders in various populations, such as individuals with Obstructive Sleep Apnea Syndrome (OSAS)^(4)^ and in studies in the aesthetics area^(5)^. It is also known that an Orofacial Myofunctional Evaluation Protocol for individuals with cleft lip and palate, adapted from the MBGR protocol^(6)^, has been expanded and validated, and validated for adults with TMD^(7)^. Regarding age range, this instrument has been applied to different life stages: children and adolescents^(8,9)^, adults^(10,11)^, the elderly^(12)^, and, more recently, infants and preschoolers^(13,14)^. On the other hand, the protocol is lengthy, which may hinder its application in situations requiring a faster assessment. Therefore, reducing the instrument's content will contribute to scientific advancement in the diagnostic process of orofacial myofunctional disorders (OMD), impacting clinical practice and facilitating its use.

To propose a reduction in the MBGR Protocol, it is necessary to comply with international guidelines that guarantee psychometric properties^(15)^, following the systematization of the methodological steps to conclude its validity evidence stages, since this standardization has enabled increasingly reliable results^(1)^. Thus, given the clinical need for a more agile and time-efficient assessment, this study aims to present a reduced version of the MBGR Orofacial Myofunctional Evaluation Protocol, addressing the following steps: validity evidence based on content and response process.

## METHOD

This is a methodological study, with a descriptive and quantitative approach, aimed at developing a reduced version of the MBGR Orofacial Myofunctional Evaluation Protocol using the adapted Delphi technique^(16)^. It should be highlighted that permission for the adaptation was obtained from the authors of the original MBGR protocol^(3)^.

### Ethical aspects

The study was approved by the Institution's Review Board (CAEE: 61170822.3.0000.5417, ID: 5.648.926). Patients and speech-language pathologists participated in the study after signing the Informed Consent Form. All participants were clearly informed about the study objectives, risks, and benefits.

### Procedures

The study followed these steps: 1) development of a preliminary reduced version of the MBGR Orofacial Myofunctional Evaluation Protocol by the authors of the original protocol; 2) validity evidence based on content; 3) validity evidence based on response processes; and 4) approval/adaptation of the final version of the reduced protocol by the authors of the original protocol.

1. Development of a reduced preliminary version of the MBGR Orofacial Myofunctional Evaluation Protocol

The authors of the original MBGR Protocol were contacted to inform them of the study's objective and to request their participation in developing the reduced version of the protocol. Next, the authors individually analyzed the protocol and proposed the exclusion or modification of items/sub-items. Following this, the authors met to discuss a proposed reduced version, with all suggestions recorded and changes defined by consensus. Thus, the preliminary reduced version of the MBGR Orofacial Myofunctional Evaluation Protocol was completed.

1. Validity evidence based on content

Eleven speech-language pathologists, designated as examiners, were invited to analyze the preliminary reduced version of the protocol, considering the following criteria: having used the original version of the protocol during their undergraduate studies in Speech-Language Pathology, and also having experience using the protocol in their professional practice.

In relation to the professional profile of speech-language pathologists, the length of their training ranged from 1 to 15 years, 54.54% had completed or were completing a residency program, 63.63% held a master's degree or were pursuing a master's degree, and 45.45% held a doctorate or were pursuing a doctorate. It is noteworthy that 100% of the professionals had some experience applying MBGR in its original version, according to the inclusion criteria adopted.

The preliminary reduced version of the MBGR Orofacial Myofunctional Evaluation Protocol was uploaded into the Google Forms® platform, so that, for each of the items/sub-items, a Likert scale with a score from one to four was used to analyze its relevance/representativeness: 1 = not relevant/not representative, 2 = needs major revision to be relevant/representative, 3 = needs minor revision to be relevant/representative, 4 = relevant/representative^(17)^. At the end of each item, there was a space to add the reason for the answer, give suggestions, or leave a comment.

After the examiners accepted the invitation, a link to access the Google Forms® platform to conduct an initial analysis was sent to them. The responses from the analyses were tabulated, the comments and suggestions were categorized, and the necessary adjustments were made, resulting in the reformulated, reduced preliminary protocol. Next, the examiners were contacted again for clarification and feedback on their initial analysis, and invited to conduct a second analysis, this time of the revised, reduced preliminary version, in the same way as in the first analysis. Again, some adjustments were necessary, based on the suggestions.

Agreement between reviewers was analyzed using the Content Validity Index (CVI)^(18)^, with the calculation performed in Microsoft Excel 2019®. First, the CVI of each item was calculated individually, considering the proportion of responses with a score of 3 or 4 in relation to the total number of examiners. Next, the overall CVI of the protocol was obtained from the average of the CVI values ​​of the items evaluated in each round. Values ​​equal to or greater than 0.80 were considered indicative of the adequacy and representativeness of the items.

Following these analyses, the reduced and revised preliminary version of the protocol, incorporating the requested changes to items 1 and 2, was returned to the professionals for further analysis of the modifications/adjustments made. The suggestions were also considered for the pre-final version.

1. Validity evidence based on response processes

This procedure involved four phases and was adapted from studies on standardization, examiner training^(14)^, applicability and documented feedback^(19)^ to ensure that the simplified version meets the criteria of the original protocol^(20)^. Phase 1: Training of speech-language pathologists; Phase 2: Application of the reduced protocol; Phase 3: Suggestions immediately after the application of the protocol and comparative analysis of the reduced version with the original protocol; Phase 4: Analysis by the authors of the original protocol.

For this step, 11 patients from a Speech-Language Pathology School Clinic, diagnosed with orofacial myofunctional disorder, aged between 19 and 44 years, were selected. They were contacted by telephone to explain the objective of the study, their participation, and to check their availability.

It is noteworthy that the number of participants was determined to correspond to the number of examiners in the study, following methodological recommendations for validity evidence based on response processes^(15,19)^.

The inclusion criteria were: age compatible with the application of the protocol (children, adolescents, or adults), possibility of complete application of the MBGR reduced version, and prior identification of orofacial myofunctional alteration. All participants contacted met the inclusion criteria, agreed to participate by signing the informed consent form, and completed the protocol application in its entirety, with no dropouts or sample losses.

In phase 1, examiners were trained, with the time required for guidance measured based on the MBGR user manual^(21)^, explaining the objective of the protocol, the concept of each question, and how the reduced version would be applied. In phase 2, the reduced protocol was applied in person, with the therapy recorded on video (Intelbras camera, model iM5) to measure the protocol application time.

In phase 3, examiners received a questionnaire consisting of 17 questions about the reduced version of the protocol, to be answered immediately after the protocol was administered. There were questions related to the understanding and application of the reduced protocol (1, 3, 5, 7, 9, 11 and 13), analyzed using a Likert-type scale: 1 = strongly agree; 2 = agree; 3 = neither agree nor disagree; 4 = partially disagree; and 5 = strongly disagree. There were also questions related to the relevance/importance of the items (2, 4, 6, 8, 10, 12, and 14), also analyzed using a Likert scale: 1 = very important; 2 = important; 3 = moderate; 4 = sometimes important; and 5 = not important at all. Finally, question 15 asked examiners to indicate whether they considered the protocol extensive, question 16 asked if the protocol was applicable in clinical practice, while question 17 provided space for suggestions and comments.

Eight days later, the examiners responded online to the same questionnaire, now comparing the original and reduced versions of the MBGR Protocol. The objective here was to verify the understanding and importance of the items, as well as to obtain suggestions for change, by analyzing the entire reduced protocol. The responses were tabulated and subjected to qualitative analysis, through the description of the responses, and to quantitative analysis, with a comparison between the original and reduced versions of the protocol. Initially, the distribution of the data was assessed for normality. For variables with a normal distribution, the paired t-test was used; when normality was not met, the Wilcoxon test for paired samples was used. A significance level of 5% was adopted.

In Phase 4, two of the authors of the original protocol analyzed the final version of the reduced protocol, and minor adjustments, identified from their experiences in teaching, research, and clinical practice, were necessary. In this process, it was also decided to update the Figure Boards and the Speech Assessment Recording Chart, contained in the article “Tongue frenulum evaluation protocol”^(22)^, with prior authorization from the journal. The new illustrations were developed by the authors with the aid of the ChatGPT Artificial Intelligence tool (GPT-5 model, OpenAI), preserving the original proposal and ensuring image standardization. This material can be used during the application of the reduced version of the MBGR Protocol, specifically in the evaluation of the "Orofacial Functions" item, for the "Speech" sub-item.

## RESULTS

1. Development of the preliminary reduced version of the MBGR Protocol

The original MBGR Protocol contained 10 pages and 8 items, which were reduced to 5 pages and 6 items in the reduced version. About the overall score, the maximum value indicating the worst condition was 353, and it was reduced to 137. For the reduced version, some items or sub-items were excluded.

The final score can range from 0 to 137 points, with lower scores reflecting better orofacial myofunctional balance. Therefore, clinical interpretation should consider the obtained value in conjunction with the examiner's analysis.

Item 1, Body Posture, was removed as it did not provide significant data for the reduced version. In item 2, Measurements of Face, Mandibular Movements, and Occlusion, the sub-items were unified, and measurements considered "extra" for a reduced evaluation were removed, resulting in 9 measurements. The 3 measurements with the caliper were maintained to ensure the accuracy of the results in a brief evaluation.

Concerning item 3, Extraoral Examination, in relation to the face, the sub-items Facial Type and Subjective Facial Analysis were removed, as these analyses only require observation by the evaluator, while Facial Proportion, Facial Symmetry, and Facial Pattern were retained. For the Lips, the sub-items were maintained, with a change in the response option for the Lower Lip Shape: three more general options to maintain efficiency. The Masseter sub-item was excluded, as it is considered a less important assessment among the others that were retained.

In item 4, Intraoral Examination, its sub-items were maintained with some modifications: adequate or altered response option for Lips and Cheeks; exclusion of the analysis of Tongue Symmetry and Height; as well as the Coloration for the Palatine Tonsils and Midline, Disocclusion Guide, and Use of Appliance in Occlusion.

In item 5, Mobility, the sub-items were maintained, but some response possibilities were removed, leaving only 3 options. Item 6, Sensitivity, was excluded because, for a brief assessment, there is no benefit in having a very specific item for diagnosis. As for item 7, Tone, the assessments were maintained in general.

In item 8, Orofacial Functions, for Breathing, the sub-item Type was removed and Nasal Flow was modified; for Chewing, the following were removed: Number of cycles, Noisy mastication, Unexpected muscle contractions, and Asking the patient; in Swallowing, only Habitual liquid swallowing was maintained; for Speech, the Phonetic/Phonological Aspect was modified, remaining: Omission, Substitution, Distortion, Tongue Projection, and Articulatory Point Exchange, with the option of Absent or Present response, excluding the sub-items Therapeutic Test, Velopharyngeal Function, and Voice, the latter being added to the General Aspects sub-item and, specifically for General Aspects, the sub-items Lip and Tongue Movements, Tongue Position in Speech, and Intonation were removed; finally, the Motor Coordination in Speech sub-item was reduced to only assess the speed and rhythm of “PATAKA”.

1. Validity evidence based on content

After the initial analysis by the examiners, the items that received a score of 4 or 5 from at least two examiners were modified. These items were: Measurements of Face, Mandibular Movements, and Occlusion; Extraoral Examination (Face); Intraoral Examination (Lips, Cheeks, Palate, Tonsil and Occlusion); Mobility (Mandible); Orofacial Functions (breathing, general aspects and motor coordination in speech). The remaining items were maintained, as they received scores of 1 and 2. The CVI calculation showed a satisfactory result (Table 1).

**Table 1:** Content Validity Index results for the Reduced Version of the MBGR Protocol

**Item**		**CVI 1st analysis**	**CVI 2nd analysis**
**1 – Measurements of Face, Mandibular Movements, and Occlusion**		0.85	1
	Face	0.71	1
**2 – Extraoral Examination**			1
	Lips	1	
	Lips	0.85	1
	Cheeks	0.71	^*^
	Tongue	1	1
**3 – Intraoral Examination**	Palate	0.85	0.85
	Palatine tonsils	0.85	1
	Teeth	1	1
	Occlusion	0.85	1
	Lips	1	1
	Tongue	1	0.85
**4 - Mobility**			
	Soft palate	1	1
	Mandible	0.71	0.85
**5 - Tone**		1	1
	Breathing	0.85	0.85
	Chewing	1	1
	Swallowing	1	1
**6 – Orofacial Functions**			
	Speech	1	1
	General aspects	0.71	1
	Motor Coordination in Speech	0.71	*
**TOTAL**		18.65 / 21 = **0.88**	16.55/19 = **0.87**

**Caption:** CVI = Content Validity Index;

* item excluded based on the evaluators suggestions

In the second analysis, of the 19 items, 15 received scores of 1 and 2, and only four received a score of 4: Intraoral Examination (Palate), Mobility (Tongue and Mandible), and Orofacial Functions (Breathing). Of the 15 items, six received a score of 1 from all examiners: Intraoral Examination (Lips, Tongue), Mobility (Soft Palate), Orofacial Functions (Chewing, Speech, and General Aspects), while eight items received scores between 2 and 3: Facial Measurements, Mandibular Movements, and Occlusion, Extraoral Examination (Face), Intraoral Examination (Lips, Palatine Tonsils, Teeth, and Occlusion), Mobility (Lips), Tone, and Orofacial Functions (Swallowing). Of the 19 items, 15 showed perfect agreement, with a CVI=1 (Table 1).

The overall final CVI score for the protocol was 0.87 (87%), above the minimum value considered acceptable.

1. Evidence based on response process

Phase 1 results showed a minimum time of 20 minutes and a maximum of 62 minutes, with a median of 56 minutes for examiner training, a mean of 51.72 minutes, and a standard deviation of ±12.37. Regarding the time required to apply the reduced protocol (phase 2), a minimum of 34 minutes and a maximum of 64 minutes were observed, with a median of 40 minutes, a mean of 42.45 minutes, and a standard deviation of ±11.83, demonstrating greater clinical efficiency in the application of the reduced MBGR protocol.

For phase 3, concerning comprehension and application (questions 1, 3, 5, 7, 9, and 11), the comparison of the scores assigned by the examiners for both versions (reduced and original) of the MBGR Protocol, using the Wilcoxon and paired t-tests, showed no difference (Table 2). This result indicates that the examiners were able to understand and apply the items: Measurements of Face, mandibular movements and occlusion; Extraoral examination, Intraoral examination; Mobility; Tone and Orofacial Functions in both protocols.

**Table 2:** Analysis of the evaluators’ responses regarding the understanding and application of the reduced and original versions of the MBGR Protocol for the following items: Measurements of Face, Mandibular Movements, and Occlusion; Extraoral Examination; Intraoral Examination; Mobility; Tone; and Orofacial Functions

	n	Median	25%	75%
P1 - Reduced	11	1.000	1.000	1.000
P1 - Original	11	1.000	1.000	2.000
P3 - Reduced	11	1.000	1.000	2.000
P3 - Original	11	1.000	1.000	2.000
P5 - Reduced	11	1.000	1.000	1.000
P5 - Original	11	1.000	1.000	2.000
P9 - Reduced	11	1.000	1.000	1.000
P9 - Original	11	1.000	1.000	2.000
P11 - Reduced	11	1.000	1.000	2.000
P11 - Original	11	1.000	1.000	2.000
	**n**	**Mean**	**Standard Deviation**	**SEM**
P7 - Reduced	11	1.273	0.467	0.141
P7 - Original	11	1.455	0.934	0.282
Difference		-0.182	0.874	0.263

**Caption:** n = number of evaluators; SEM = *Structural Equation Modeling*

Concerning relevance/importance (questions 2, 4, 6, 8, 10, 12, and 14), different results were obtained. In question 2 (measurements of the face, mandibular movements, and occlusion), for the reduced version, 64% of examiners rated this item as very important, and for the original version, there was variation in the assessment: 55% classified it as very important or important, 18% as moderate, and 27% as sometimes important. In question 12 (Orofacial Functions), 91% of examiners rated this item as very important or important in both versions, while 9% rated it as moderately important in the reduced version and 9% as sometimes important in the original version. The Wilcoxon test showed no difference, indicating that examiners classify the items Measurements of Face, Mandibular Movements, and Occlusion, as well as Orofacial Functions, as important for both protocols (Table 3).

**Table 3:** Analysis of the evaluators’ responses regarding the relevance/importance of the items Measurements of Face, Mandibular Movements, and Occlusion; Extraoral Examination; Intraoral Examination; Mobility; Tone; and Orofacial Functions in the reduced and original versions of the MBGR Protocol

	n	Median	25%	75%	
P2 - Reduced	11	1.000	1,000	3,000	
P2 - Original	11	1.000	1,000	4,000	
P12 - Reduced	11	1.000	1,000	1,000	
P12 - Original	11	1.000	1,000	1,000	
	**n**	**Mean**	**Standard Deviation**	**SEM**	**p - value**
P4 - Reduced	11	1.545	0.522	0.157	
P4 - Original	11	3.000	1.000	0.302	**p< 0.001**
Difference		-1.455	0.934	0.282	
P6 - Reduced	11	1.273	0.467	0.141	
P6 - Original	11	2.636	1.690	0.509	**p< 0.001**
Difference		-1.364	1.804	0.544	
P8 - Reduced	11	1.636	1.027	0.31	
P8 - Original	11	2.000	1.095	0.33	p > 0.05
Difference		-0.364	0.924	0.279	
P10 - Reduced	11	1.545	0.934	0.282	
P10 - Original	11	1.636	0.809	0.244	p > 0.05
Difference		-0.0909	0.944	0.285	

**Caption:** n = number of evaluators; SEM = *Structural Equation Modeling*

In questions 4 (Extraoral Examination) and 6 (Intraoral Examination), the paired t-test analysis showed that the average score assigned by the examiners to the reduced version was lower (p<0.001), demonstrating that these items were relevant/important; unlike the results of questions 8 (Mobility) and 10 (Tone), where the paired t-test did not show a difference between the average scores of the original and reduced versions (Table 3).

Regarding the protocol summary sheet and the suggested image recording script (questions 13 and 14), most examiners suggested their exclusion, justified by the impact on application time and the absence of reference values ​​for comparison. Nevertheless, it was decided to retain the summary and script, aiming for future validation with the definition of these parameters.

The results of question 15, about the length of the protocols, showed that, for the reduced version, 73% totally (36.5%) or partially (36.5%) disagreed that it was extensive, 9% neither agreed nor disagreed, and 18% considered it extensive; about the original version, 73% totally (55.5%) or partially (36.5%) agreed, and 9% disagreed that it was extensive. These results demonstrated, using the Wilcoxon test, that the reduced version is not extensive compared to the original version (Table 4).

**Table 4:** Analysis of the evaluators’ responses regarding the length and applicability of both versions of the MBGR Protocol

	n	Median	25%	75%
P15 - Reduced	11	2.000	1.000	3.000
P15 - Original	11	5.000	4.000	5.000
P16 - Reduced	11	2.000	1.000	2.000
P16 - Original	11	4.000	3.000	4.000

**Caption:** n = number of evaluators

In question 16, examiners were asked about the applicability of the reduced version in clinical practice, with 91% agreeing totally (45.5%) or partially (45.5%) and 9% disagreeing; regarding the original version, 18% agreed, 27% neither agreed nor disagreed, and 55% disagreed partially (45.5%) or totally (9.5%). Thus, these results show that the majority of examiners agreed totally or partially that the reduced version is applicable in clinical practice, while 55% disagreed totally or partially that the original version is applicable in clinical practice, this difference being observed in the Wilcoxon test (Table 4).

Another analysis performed was a comparison of the comprehension, application, and importance of each item, 8 days after the application of the reduced version. Positive comments were obtained from the examiners, reaffirming the importance of the items in the reduced version (Chart 1), as well as suggestions (Chart 2).

**Chart 1:** Positive comments from the evaluators regarding the reduced version

**MEASUREMENTS OF FACE, MANDIBULAR MOVEMENTS, AND OCCLUSION**	
Understanding and application	1. Important for understanding facial proportions and structural limitations.
	2. The explanations for each item are important as reminders in case of uncertainty or forgetting.
	3. The instructions are clear and understandable.
Importance	4. This item indicates what can be expected in the subsequent assessment items.
	5. The measurements of mandibular movements and occlusion contribute to the speech-language pathology diagnosis.
**EXTRAORAL EXAMINATION**	
Understanding and application	1. Helps investigate characteristics that indicate structural and functional alterations.
	2. The instructions are clear and understandable.
Importance	3. Facial pattern, lip posture, and mucosa contribute to the speech-language pathology diagnosis.
	4. Important to confirm or not the patient’s report regarding breathing.
	5. It is important and provides indications of the tone of the structures.
	6. Extraoral conditions may influence functions and, therefore, I consider their assessment important even in a reduced version.
**INTRAORAL EXAMINATION**	
Understanding and application	1. All items are clear.
	2. The instructions are clear and understandable.
	3. Assesses intraoral characteristics and facilitates the understanding of functionality.
	4. Important, especially for assessing the patient’s oral health and referring them to other professionals in cases of suspected alterations, such as cancer or poor oral hygiene.
	5. This item is essential.
**MOBILITY**	
Understanding and application	1. I understand/agree with the inclusion of the four mobility items.
	2. The instructions are clear and understandable.
	3. An essential item.
**TONE**	
Understanding and application	1. The instructions are clear and understandable.
Importance	2. It is possible to relate this item to other assessed aspects, such as extraoral examination, mastication, and mobility.
	3. I believe so, since functional alterations may occur in the presence of abnormalities.
**OROFACIAL FUNCTIONS**	
Understanding and application	1. The instructions are clear and understandable.
Importance and evaluation	2. After assessing the structures, it is necessary to understand how they are functioning; therefore, this item is extremely important.
	3. Indispensable.

**Chart 2:** Evaluators’ suggestions regarding the reduced version

**MEASUREMENTS OF FACE, MANDIBULAR MOVEMENTS, AND OCCLUSION**		**MF**
Understanding and application	1. It would be important to specify which value should be considered in cases of discrepancy.	-
Importance	2. Considering the reduced version, perhaps only one measurement should be performed.	YES
	3. Measurements of mandibular movements and occlusion contribute to the speech-language pathology diagnosis, whereas facial measurements do not.	NO
	4. It would be interesting to include a small image illustrating TV and TH alongside, or instead of, the description to help professionals remember the measurements.	YES
	5. Mandibular movements are important; facial measurements may be more relevant in specific cases.	NO
	6. I believe this item could be omitted in clinical practice.	NO
**EXTRAORAL EXAMINATION**		
Understanding and application	1. It would be interesting to specify that facial proportion is obtained from the facial measurements data, similarly to the previous descriptions. Likewise, “concave” and “convex” are, in my opinion, difficult terms that are not very helpful.	YES
Importance	2. This item may be better applied in specific cases rather than routinely.	NO
**INTRAORAL EXAMINATION**		
Importance	1. I would remove only the **“Number of teeth”** item.	YES
	2. The intraoral examination could be simplified, for example, by including only an “oral cavity” section in which altered aspects found during the assessment could be scored generally, without listing normal items, while still observing all structures.	NO
	3. I believe that **counting teeth** is unnecessary in the reduced version.	YES
**MOBILITY**		
Understanding and application	1. Correct only the letter “E” for the left side.	YES
Importance	2. This becomes secondary in the presence of functional alterations. If function is altered, then it is worth investigating further.	NO
	3. I believe that lip alternation, as well as **soft palate and mandibular mobility**, are not important unless the patient presents a complaint or condition related to these aspects, in which case they could be included under “observations.”	NO
	4. I believe that the soft **palate (and mandible)** item is unnecessary, considering this is a reduced version.	NO
**TONE**		
	No suggestions.	
**OROFACIAL FUNCTIONS**		
Understanding and application	1. In the nasal breathing possibility section, indicate that timing should be recorded either in writing or with a clock symbol.	YES
Importance	2. The patient presented alterations in solid swallowing that were not recorded because there was no specific field for this observation.	YES
	3. In the speech section, I would keep the **spontaneous speech** sample item.	YES
	4. These are the most important aspects to be assessed. It would be even more valuable to include a broader speech sample for evaluation (e.g., counting days of the week and **spontaneous speech**).	YES
	5. Since this is a reduced version, I believe the nasal breathing possibility items may not be as important, with only nasal airflow being assessed. Regarding mastication, everything is fine.	NO

**Caption:** MF = Modification Fulfilled

Analyzing the examiners' comments, it was found that some suggested changes to the reduced version, and the suggestions that were not accepted were duly justified. About the modifications made to the reduced version, it is understood that the suggestions were necessary to improve the quality of the protocol.

Regarding the item "Measurements of face, mandibular movements, and occlusion," the first suggestion was not accepted, as the second was. Therefore, the 2nd and 3rd measurements were excluded, without needing to record the value in case of discrepancy. The third suggested alteration was not possible, considering that facial measurements also contribute to the speech-language pathology diagnosis. The fourth suggestion, however, was accepted and applied to other measurements, with images added to facilitate and remind the reader of how to perform the measurements. The last suggestion was not considered, as this item cannot be entirely excluded, since this analysis is of great importance in the structural evaluation.

In the extraoral examination, the first suggestion was considered, aiming to facilitate the application of the reduced version in a shorter time, but the second was not considered, since the extraoral examination is part of the structural evaluation.

In relation to the intraoral examination, two different professionals suggested excluding the tooth count, which was accepted; images of the deciduous and permanent dentition were added for illustration. On the other hand, the suggestion of a general evaluation, without mentioning the item analyzed, was not accepted, as the protocol aims to standardize the evaluation.

Regarding mobility, the suggestion to correct the letter L to represent the left side was considered, but the others could not be adopted. The muscle tone item received no suggestions.

For orofacial functions (breathing, chewing, swallowing, and speech), five suggestions were analyzed. The first was accepted and referred to inserting a clock symbol to indicate the need to time the "possibility of nasal use," also including a symbol of a glass with liquid, indicating the need to maintain a volume of water in the mouth during the test, aiming for practicality in applying the reduced version of the protocol in a shorter amount of time. As it was observed that the professional suggested images to facilitate the application, functioning as a reminder, this suggestion expanded to other sections in the protocol, where images could be added to further facilitate the application. The second suggestion, to add a specific field, was not accepted, as the extra data can be noted in the "observation" field. The third and fourth suggestions, to maintain samples of spontaneous and automatic speech in the speech assessment, were considered, and the information that speech language pathologists can use both aspects in speech assessment was even added to the protocol. Finally, the fifth suggestion, to exclude the possibility of nasal use, was not accepted, given that this aspect represents an important item for guiding the therapy of patients with oronasal breathing.

In Phase 4, the final version of the reduced protocol was presented to the authors of the original protocol, who considered most of the adjustments adequate. In the section "Measurements of face, mandibular movements, and occlusion," the words third and "(lips), written with lowercase initials, were corrected to uppercase initials; the abbreviations UL (Upper Lip) and LL (Lower Lip) were also included. In the section "Extraoral Examination," the term "length of the upper lip" was excluded and replaced by two others: "exposure of the upper incisors at rest" and "UL/LL ratio."

In the Orofacial Functions section, the aspects "adequate," "reduced unilateral," and "reduced bilateral" were included for breathing in the "nasal flow" sub-section.

For chewing, the sub-item "efficiency" was added, also including the aspects "alternating bilateral" and "alternating unilateral". In swallowing, which previously was restricted to the evaluation of liquids, the scope was expanded to also include solids, being reformulated as "habitual swallowing of solids and liquids". Consequently, the sub-item "liquid retention" was replaced by "food retention". Furthermore, new items were included, covering the assessment of head movements and the contraction of the orbicularis oris and mentalis muscles.

About speech, the sub-item "change of articulatory point" was excluded, and the sub-item "tongue projection" was replaced with "lingual interdentalization".

Regarding the picture boards, it was decided to replace the image of an old telephone with that of a cell phone, aiming to update the image due to technological changes and ensure greater recognition by children, who have difficulty identifying the previous telephone model. Regarding the Speech Assessment Recording Chart, the term ‘patient production’ was replaced by ‘present alteration’ to align the terminology with the expected phonetic aspect in the field of OMT. Finally, the 50 words from the original list were maintained^(22)^.

Based on the modifications made, the final version of the MBGR Reduced Protocol (MBGR-r) was obtained, as shown in Appendix A.

## DISCUSSION

The development of the reduced version of the MBGR Orofacial Myofunctional Evaluation Protocol followed the steps of validity evidence. A key innovation of this study is the use of Artificial Intelligence (ChatGPT, GPT-5 model, OpenAI) to update and standardize the illustrations for the reduced version of the MBGR Protocol. This approach contributed to greater visual uniformity of stimuli and represents an emerging interface between technology and health, expanding possibilities for the development and modernization of clinical instruments in the Speech-Language Pathology area.

In updating the illustrations, the aim was to fully preserve the structure, organization, and references of the original figures, ensuring coherence with their clinical purpose. The modifications made were essentially graphic in nature, to standardize visual stimuli and reduce elements that could induce age, generation, or personal identification, making the images more neutral and timeless, especially regarding the "Telephone" figure. Therefore, the update did not imply a conceptual replacement of the figures, but rather a visual adaptation aligned with technological and communicational transformations.

For standardized use of the protocol, it is essential to adhere to the validation procedures, respecting the guidelines that ensure its psychometric properties, as recommended by the literature^(15)^. The CVI results indicated that the reduced protocol has good representation of the essential items^(21)^ for orofacial myofunctional evaluation, with specific adjustments being necessary to optimize the applicability and clarity of the items. Continuous analysis and subsequent validation of the protocol, along with the implementation of these modifications, are important steps to ensure that the instrument meets the needs of professionals in the area and provides more accurate and efficient diagnoses^(16)^.

The overall CVI score of 87% indicates that the reduced MBGR Protocol has obtained initial evidence of validity for clinical use^(21)^, and also shows that the review process was conducted rigorously and collaboratively, with the integration of experts and adjustments based on evidence ^(14,19,20)^. These results suggest that the protocol can be an effective tool for assessment, meeting the needs of practicality without compromising diagnostic quality. From the results, it can be inferred that the reduced MBGR Protocol has greater applicability in the daily clinical practice of speech-language pathologists compared to the original version of the protocol. The reduction in application time makes the reduced version more efficient, facilitating its administration and making it more practical for use in routine clinical settings^(23)^. The literature highlights the importance of this trend, since the use of lengthy protocols can be limited in health services due to their complexity and response load, which makes the adoption of shorter and more objective versions essential^(24)^.

The incomplete exclusion of measurements of face, mandibular movements, and occlusion section reinforces the need for a detailed structural assessment, allowing for a more precise and individualized approach for each patient^(25)^. Maintaining normative and expected values ​​is fundamental for detecting deviations from normality^(26)^, as they may be associated with skeletal, functional and neuromuscular problems^(25)^ and, therefore, for understanding the relationship between muscle balance and orofacial structures with stomatognathic functions^(3)^. The inclusion of images to illustrate facial measurements, mandibular movements, and occlusion reflects a concern for the standardization and reproducibility of the protocol, since the literature indicates that the use of visual aids helps minimize measurement errors and improves the understanding of the evaluators, positively impacting the reliability of the results^(27)^.

The non-complete exclusion of the extraoral examination section reinforces the importance of evaluating structures such as the lips and face, when analyzing aspects assessed such as the facial pattern, which assists in the investigation of cases of patients with craniofacial alterations that may predict the risk of Orofacial Myofunctional Disorder^(26)^ and with malocclusion, Angle class II and class III and their dental and skeletal components^(25)^.

Regarding the intraoral examination, the rejection of the suggestion for a more general intraoral assessment reinforces the importance of standardizing the protocol. It is understood that a standardized assessment allows for greater reliability in data collection and avoids subjective interpretations that could compromise diagnostic accuracy^(2)^. Literature suggests that detailed assessments favor the early identification of structural and functional alterations, essential for speech-language pathology practice, highlighting the interaction between form and function of the speech organs, which, in performing their motor and neuromuscular functions, ensure the harmony of the craniofacial complex^(28)^.

The mobility section did not take into account data on exclusion suggestions. Analysis of lip, tongue, and jaw mobility tests allows for the identification of muscular compensations not involved in the requested movement; an example is the contraction of the platysma muscle during mandibular laterality, as a compensation in cases of muscular and articular dysfunction^(25)^. Assessing mobility is crucial for observing muscle activity, which is directly related to orofacial functions and craniofacial growth^(2)^. The mobility of the soft palate is fundamental for detecting possible breathing difficulties during sleep^(4)^, and specifically, limitations in mandibular movements are also important in cases of radiotherapy in the area, in the case of cancer patients, and can cause difficulties in performing oral functions^(29)^.

The modifications to the orofacial function sections aim to balance practicality with the maintenance of essential aspects for speech-language pathology assessment^(1)^. The suggestions that were accepted, such as the optimization and standardization of the protocol, are aligned with the literature^(23)^. However, the suggestions that were not implemented were preserved due to their importance in diagnosis and therapeutic guidance.

The approval of the final protocol by the authors of the original MBGR Protocol validates the quality of the study, demonstrating that the modifications respected the principles and objectives of speech-language pathology assessment. However, a limitation of the study is the age range of the patients evaluated, as they were between 19 and 44 years old, considering that the original MBGR was proposed for use from 6 years of age onwards. Furthermore, although the number of evaluators is in line with methodological recommendations for validation studies, this characteristic can also be considered a limitation of the study, since a larger number of speech-language pathologists could broaden the diversity of perceptions and clinical experiences.

It is also worth noting that the examiners' prior experience with the original version may have favored the observed applicability, since familiarity with the protocol's structure and criteria tends to facilitate its use, limiting the identification of difficulties that could emerge in contexts with professionals without prior experience. Finally, it is understood that the results obtained indicate the need and relevance of continuing with the remaining validation stages of the reduced version, following the planned methodological path, to expand psychometric evidence and consolidate its use in different clinical contexts.

Considering its more concise structure and easier applicability, the MBGR reduced version may have potential for use in screening or teleassessment contexts, especially in scenarios that require assessments in a shorter time. Although such applications still require specific study, this possibility could broaden the clinical reach and performance of the instrument in different real-world speech-language pathology contexts.

In summary, the results demonstrate the feasibility and relevance of the reduced version of the MBGR Protocol, contributing to a more agile, objective, and standardized speech-language pathology assessment. This study strengthens the OMT area by offering an instrument with initial evidence of validity and functionality, aligned with clinical demands and evidence-based practice. Future studies could deepen psychometric analyses, establish normative parameters, and expand its use in different contexts, consolidating its potential as a reference tool in the area.

## CONCLUSION

The MBGR Protocol was reduced from 36 items to 19 items in total, with the worst score decreasing from 353 to 134, respectively. In the overall assessment, the reduced protocol achieved a CVI of 0.87, higher than the acceptable value. The examiners' suggestions were considered, items were removed, thus improving and optimizing the use of the MBGR reduced protocol. This study provided validity evidence based on content and response processes, resulting in the final version of the protocol with a shorter implementation time.

Although the clinical phase, comprised of 11 patients, offers evidence of applicability, its small size limits the generalizability of the findings. Therefore, it is recommended that the validation steps continue, including analyses of construct validity, criterion validity, and diagnostic accuracy, to enhance the psychometric robustness and consolidate the use of the protocol in different clinical contexts for the diagnosis of orofacial myofunctional disorders.

## Figures and Tables

**Figure:**
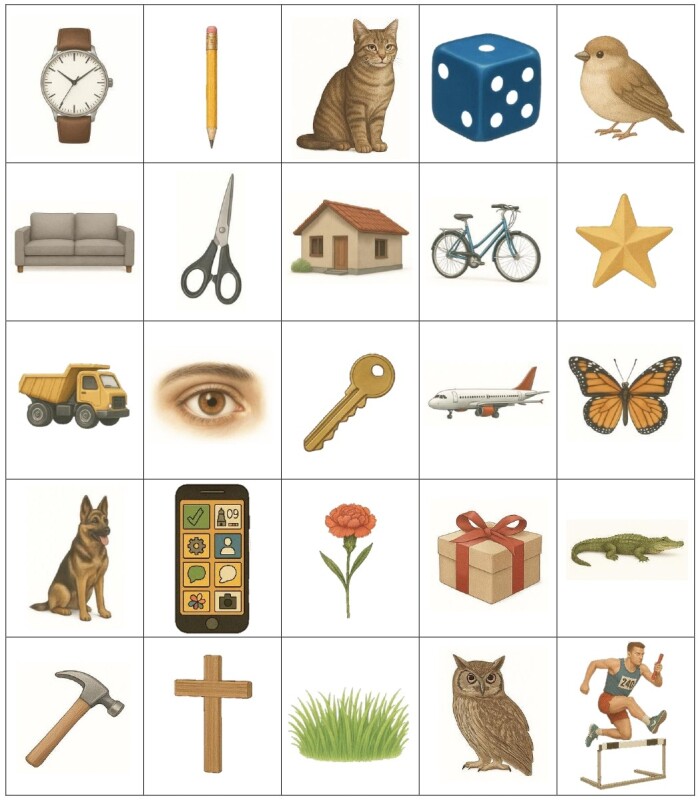
PICTURE BOARD FOR SPEECH ASSESSMENT – PART 1 (PROPOSED FOR BRAZILIAN PORTUGUESE SPEAKERS)

**Source:** Tongue frenulum evaluation protocol^(22)^

**Figure:**
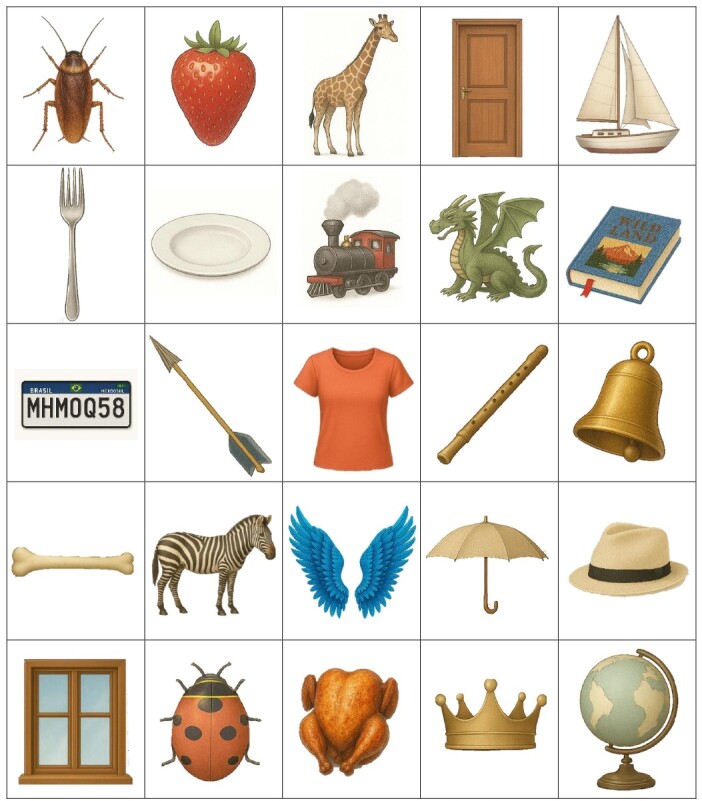
PICTURE BOARD FOR SPEECH ASSESSMENT – PART 2 (PROPOSED FOR BRAZILIAN PORTUGUESE SPEAKERS)

**Source:** Tongue frenulum evaluation protocol^(22)^

**Note:** Board updated by the authors, with images generated by Artificial Intelligence (AI), based on the original version

## Appendix A Orofacial Myofunctional Evaluation Protocol – MBGR REDUCED (MBGR-r) ***Marchesan IQ, Berretin-Felix G, Genaro KF, Rehder MI***

Name:____ MRN.:____ Date of Examination:___/___/___Age:____DOB:___/__/____Weight:____lb Height:____ft. in BMI:____(weight[lb]/height[ft]^2^x703)

1. MEASUREMENTS OF FACE, MANDIBULAR MOVEMENTS, AND OCCLUSION

Face, occlusion, and mandibular movements

**Table:**

		**Measurement** (in)
	Middle third of the face *(glabella^1^ to subnasal ^2^)*	
	Lower third of the face *(subnasal ^2^ to gnathion^5^)*	
	Upper Lip (UL) *(subnasal ^2^ to the lowest point of the upper lip^3^)*	
	Lower Lip (LL) *(from the uppermost point of the lower lip ^4^ to the gnathion^5^)*	
	Overbite (OB) *(with the teeth in occlusion, mark the incisal edge of the upper incisors on the vestibular of the lower incisors and measure the distance from the marking to the incisal edge of the inferior incisors; in the open bite, measure the distance between the incisal edges of the upper and lower incisor teeth on the vertical plane, and the result obtained will be negative)*	
	Overjet (OJ) *(measure the distance between the incisal edges of the upper and lower incisors on the horizontal plane)*	
	Right mandibular laterality^6^ *(mark the midline of the upper dental arch on the lower arch, move the mandible to the right and hold, mark the midline of the upper dental arch again on the lower arch, return to the resting position and measure the distance between the two markings )*	
	Left mandibular laterality^6^ *(mark the midline of the upper dental arch on the lower arch, move the mandible to the left and hold, mark the midline of the upper dental arch again on the lower arch, return to the resting position and measure the distance between the two markings )*	
	Maximum active interincisional distance (MAID) *(from the upper central or lateral incisor to the lower incisor with the mouth fully open)*	

**2. EXTRAORAL EXAMINATION [ ]** Sum of points for face and lips *(best result = 0 and worst = 14* )

**Face [ ]** (*best result = 0 and worst = 3*) *(In terms of facial proportions, answer according to measurements of the thirds of the face)*

**Table:**

Facial proportion:	(0) similar middle and lower third	(1) lower third	(2) long lower third
Facial pattern:	(0) pattern I (straight)	(1) pattern II (convex)	(1) pattern III (concave)

Observation:_________________________________________________________________________________________________________

**Lips [ ]** (*best result = 0 and worst = 11) (In terms of lip proportion, answer according to UL and LL measures)*

**Table:**

Habitual posture:		(0) closed		(1) closed with tension		(2) sometimes opened, sometimes closed	
		(2) parted		(2) closed with dental contact		(3) opened	
Lower (shape):		(0) normal		(1) with mild eversion		(2) with accentuated eversion	
Exposure of upper incisor teeth at rest:			(0) covers ⅔ of the incisors		(1) covers more than ⅔		(2) covers less than ⅔
UL/LL ratio:	(0) UL ½ of LL measurement				(1) UL > ½ of LL measurement		(2) UL < ½ of LL measurement
External mucosa:	(0) normal			(1) moist	(1) dry		(2) lesioned

Observation:_________________________________________________________________________________________________________

**3. INTRAORAL EXAMINATION [ ]** Sum of points for lips, tongue, palate, tonsils, teeth, and occlusion.

(best result = 0 and worst = 36)

**Lips [ ]** (*best result = 0 and worst = 2*)

**Table:**

Internal mucosa:	(0) adequate	(1) altered (describe):
Upper frenulum:	(0) adequate	(1) altered (describe):

Observation:_________________________________________________________________________________________________________

**Tongue [ ]** *(best result = 0 and worst = 16) (more than one option can be selected for some aspects, and all of them must be added together to obtain the score.)*

**Table:**

**Habitual posture:** ❒ not observable (1) on the floor		(1) low tip and high back (1) interdental
**Width:** (0) adequate (1) reduced		(2) increased
**_Mucosa:_** (0) normal (1) geographic (1) tooth-marked (region):		(1) fissured (2) lesioned (region): (1) marked by orthodontic appliance (region):
**Frenulum:**	Attached: to the floor: (0) visible from the caruncles to the tongue: (0) in the middle third	(1) visible from the inferior alveolar crest (1) between the middle third and the tip (2) at the tip
	Tip shape when raising the tongue: (0) rounded	(1) square or rectangular (1) slight cleft at the tip (2) heart-shaped (3) does not rise
	Thickness: (0) thin	(0) submucosal (1) thick

Observation:_________________________________________________________________________________________________________

**Palate [ ]** (*best result = 0 and worst = 8)*

**Table:**

Hard:	Depth:	(0) adequate	(1) reduced (low)	(2) increased (high)
Width:		(0) adequate	(1) increased (wide)	(2) reduced (narrow)
Soft palate: Symmetry: Extension:		(0) present (0) adequate	(1) absent (describe): (1) long (2) short	
Uvula:	(0) adequate	(1) altered (describe):		

Observation:_________________________________________________________________________________________________________

**Palatine tonsils [ ]** (*best result = 0 and worst = 2)*

**Table:**

**Presence:** ❒ present	❒ removed	❒ not observable
**Size:** (0) adequate	(1) hypertrophy R	(1) hypertrophy L

Observation:_________________________________________________________________________________________________________

**Teeth [ ]** (*best result = 0 and worst = 1*)

**Table:**

Dentition:	deciduous	mixed dentition ρ permanent	
Dental defects:	(0) absent	(1) present (elements):	

Observation:_________________________________________________________________________________________________________

**Occlusion [ ]** (*best result = 0 and worst = 7)*

**Table:**

Angle’s Classification:	R Side: (0) Class I	(1) Class II div. 1^st^ (1) Class II div. 2^nd^ (1) Class III
	L Side: (0) Class I	(1) Class II div.. 1^st^ (1) Class II div. 2^nd^ (1) Class III
Horizontal relation:	(0) adequate [*0.03 to o.11 in*] edge-to-edge bite [*0 in*]	(1) excessive overject [*> 0.11 in*] (1) anterior crossbite [*< 0 in*]
Vertical relation:	(0) adequate [*0.03 to o.11 in*] edge-to-edge bite [*0 in*]	(1) excessive overbite [*> 0.11 in*] (1) posterior open bite R (1) anterior open bite *[< 0 in*] (1) posterior open bite L
Transverse relation:	(0) adequate	(1) posterior crossbite R (1) posterior crossbite L

Observation:_________________________________________________________________________________________________________

**4. MOBILITY [ ]** Add up the points for lips, tongue, soft palate, and jaw ( *best result = 0 and worst = 28* )

**Lips [ ]** (*best result = 0 and worst = 9*) *(keep teeth closed*)*

**Table:**

	Adequate	Minor alteration	Significant alteration	Absent
Protraction of closed lips*:	(0)	(1)	(2)	(3)
Retraction of closed lips*:	(0)	(1)	(2)	(3)
Protraction of closed lips and alternation to the right and left*:	(0)	(1)	(2)	(3)

Observation:_________________________________________________________________________________________________________

**Tongue [ ]** (*best result = 0 and worst = 9*)

**Table:**

	Adequate	Minor alteration	Significant alteration	Absent
Touch the tongue tip sequentially at the R/L commissures and U/L lip.	(0)	(1)	(2)	(3)
Touch of the tongue tip on the incisive papilla:	(0)	(1)	(2)	(3)
Sucking of the tongue on the palate:	(0)	(1)	(2)	(3)

Observation:_________________________________________________________________________________________________________

**Soft palate [ ]** (*best result = 0 and worst = 4*)

**Table:**

Repeated pronunciation of the vowel "a".: ^Adequate Reduced Absent^ (0) Right (0) Left (1) Right (1) Left (2) Right (2) Left

Observation:_________________________________________________________________________________________________________

**Mandible [ ]** (*best result = 0 and worst = 6*)

**Table:**

Opening of the mouth: *(MAID + TV)*	Adequate Reduced Increased (0) (1) (1) *Expected values: children = 1.37 in to 1.96 in / adult = 1.57 in to 2.16 in*		Absent (2)
Laterality: Right: Left:	(0) (0)	(1) (1) (1) (1) *Expected values: children (6 to 12 years old ) = 0.23 in to 0.39 in / adult = 0.31 in to 0.41 in*	(2) (2)

Observation:_________________________________________________________________________________________________________

**5. TONE [ ]** *(best result = 0 and worst = 6) (perform visual observation and palpation)*

**Table:**

	Normal	Reduced	Increased
Lips:	(0) Superior (0) Inferior	(1) Superior (1) Inferior	(1) Superior (1) Inferior
Cheeks:	(0) Right (0) Left	(1) Right (1) Left	(1) Right (1) Left
Chin:	(0)	(1)	(1)
Tongue:	(0)	(1)	(1)

Observation:_________________________________________________________________________________________________________

**6. OROFACIAL FUNCTIONS [ ]** Add up the points for breathing, chewing, swallowing, and speech.

(best result = 0 and worst = 53)

**Breathing [ ]** *(best result = 0 and worst = 6)*

If altered, the origin is [ ] functional [ ] structural [ ] other:

**Table:**

**Mode:** (0) nasal (1) oronasal	(2) oral		
**Possibility of nasal use:**	(0) 2 minutes or more	(1) between 1 and 2 minutes	(2) less than 1 minute
**Nasal flow:** (0) adequate	(1) unilateral reduced	(2) bilateral reduced	

Observation:_________________________________________________________________________________________________________

**Habitual Chewing [ ]** (*best result = 0 and worst = 8*) *(whenever possible, always use the same food.)*

If altered, the origin is [ ] functional [ ] structural [ ] DTM [ ] other:________________

**Table:**

**Incision:** (0) anterior (1) lateral		(1) other: _______
**Chewing:** (0) posterior teeth (1) anterior teeth		(1) with the tongue
**Efficiency:** (0) adequate (1) altered		
**Chewing pattern:**	(0) bilateral alternated *(50% - 65%)*	(0) unilateral alternated *(50% - 65%)* (0) unilateral preferential *(66% - 75%)*
	(1) bilateral simultaneous (> 65%) (2) unilateral chronic (> 75%)	
**Closing lips:** (0) systematic (1) unsystematic		(2) absent
**Rhythm:** (0) adequate (1) slow		(1) fast

Observation:_________________________________________________________________________________________________________

**Habitual Swallowing of Solids and Liquids***(water)***[ ]** (*best result = 0 and worst = 15*)

If altered, the origin is [ ] functional [ ] structural [ ] other:___________

**Table:**

Posture of the tongue:	❒ Not observable	(0) behind the teeth	(1) against the teeth	(2) between the teeth
Containment of food:	(0) adequate	(1) inadequate solid	(1) inadequate liquid	(2) inadequate solid and liquid
Contraction of the orbicularis:	(0) absent	(1) increased solid	(1) increased liquid	(2) increased solid and liquid
Contraction of the mentalis:	(0) absent	(1) increased solid	(1) increased liquid	(2) increased solid and liquid
Movement of the head:	(0) absent	(1) present solid	(1) present liquid	(2) present solid and liquid
Volume of liquid:	(0) satisfactory	(1) reduced	(2) increased	
Liquid intake rhythm:	(0) sequential	(1) sip by sip		
Noise:	(0) absent	(1) present solid	(1) present liquid	(2) present solid and liquid

Observation:_________________________________________________________________________________________________________

**Speech [ ]** Add up the points for speech sound production and general aspects *(best result = 0 and worst = 24)*

If altered, the origin is [ ] functional [ ] structural [ ] phonological [ ] other:

**Speech sound production [ ]** *(best result = 0 and worst = 8) (to assess through spontaneous, automatic speech and naming of figures)*

**Table:**

	Absence	Unsystematic Systematic presence presence		Describe
**Omission:**	(0)	(1)	(2)	
**Substitution:**	(0)	(1)	(2)	
**Distortion:**	(0)	(1)	(2)	
**Interdentalization:**	(0)	(1)	(2)	

Observation:_________________________________________________________________________________________________________

**General aspects [ ]** *(best result = 0 and worst = 16) (More than one option can be selected for some aspects, and all of them must be added together to obtain the score.)*

**Table:**

**Saliva:** (0) swallowed (1) accumulated in the right and/or left commissure of the mouth (1) accumulated on the lower lip (2) spit
**Opening of the mouth:** (0) adequate (1) reduced (1) increased
**Position of the tongue:** (0) adequate (1) on the floor of the mouth (1) retracted (1) forwarded
**Movement of the mandible:** (0) adequate trajectory (1) right deviation (1) left deviation (1) retraction
**Articulation:** (0) precise (1) unsystematic imprecision (2) systematic imprecision
**Rate of speech:** (0) adequate (1) increased (1) reduced
**Pneumo-phono-articulatory coordination:** (0) adequate (1) altered:
**Resonance:** (0) balanced (1) reduced nasal use (1) excessive nasal use: ❒ mild ❒ moderate ❒ severe (1) excessive laryngeal use (1) excessive pharyngeal use: ❒ mild ❒ moderate ❒ severe

Observation:_________________________________________________________________________________________________________

Data collected from examinations:

Requested examinations (reason):

Speech-Language Pathology diagnosis:

**Prognosis:** ❒ good ❒ guarded ❒ poor

Therapeutic plan:

Referral to other professionals (reasons):

SUMMARY OF THE OROFACIAL EVALUATION EXAM - MBGR REDUCED (MBGR-r) ***Marchesan IQ, Berretin-Felix G, Genaro KF, Rehder MI***

**Table:**

**EXTRAORAL EXAMINATION (best result = 0 and worst = 14)**	**[ ]**
Face (best result = 0 and worst = 3)	[ ]
Lips (best result = 0 and worst = 11)	[ ]
**INTRAORAL EXAMINATION (best result = 0 and worst = 36)**	**[ ]**
Lips (best result = 0 and worst = 2)	[ ]
Tongue (best result = 0 and worst = 16)	[ ]
Palate (best result = 0 and worst = 8)	[ ]
Palatine tonsils (best result = 0 and worst = 2)	[ ]
Teeth (best result = 0 and worst =1)	[ ]
Occlusion (best result = 0 and worst = 7)	[ ]
**MOBILITY (best result = 0 and worst = 28)**	**[ ]**
Lips (best result = 0 and worst = 9)	[ ]
Tongue (best result = 0 and worst = 9)	[ ]
Soft palate (best result = 0 and worst = 4)	[ ]
Mandible (best result = 0 and worst = 6)	[ ]
**TONE (best result = 0 and worst = 6)**	**[ ]**
Lips (superior + inferior) (best result = 0 and worst = 2)	[ ]
Cheeks (right+ left) (best result = 0 and worst = 2)	
Chin (best result = 0 and worst = 1)	[ ]
Tongue (best result = 0 and worst = 1)	[ ]
**OROFACIAL FUNCTIONS (best result = 0 and worst = 53)**	**[ ]**
Breathing (best result = 0 and worst = 6)	[ ]
Chewing (best result = 0 and worst = 8)	[ ]
Swallowing (best result = 0 and worst = 15)	[ ]
Speech (best result = 0 and worst = 24)	[ ]
**TOTAL SCORE** (0 – 137 points)	

Speech-Language Pathologist: ______________________________________________________________________________________________________________________

CHECKLIST FOR PATIENT’S IMAGES – MBGR REDUCED (MBGR-r)

Static Images

**Table:**

**Face:*** ❒ front		❒ lower third of the front	❒ right profile at rest
**Teeth:** ❒ superior arch		❒ inferior arch	
**Occlusion:** ❒ anterior		❒ right side	❒ left side
**Tongue:**	❒ at rest on the floor of the mouth ❒ protruded		
	❒ frenulum (the tongue is raised within the oral cavity without touching the palate.)		

*With correction of head posture

dynamic images

**Table:**

Mobility:*	❒ lips	❒ tongue	❒ mandible ❒ soft palate
Chewing:	❒ habitual	❒ questions	
Habitual swallowing:	❒ solid	❒ liquid	
Speech:	❒ spontaneous	❒ automatic	❒ naming of figures

*Request three repetitions of each movement.

**Note:** Board updated by the authors, with images generated by Artificial Intelligence (AI), based on the original version

SPEECH ASSESSMENT RECORDING CHART (PROPOSED FOR BRAZILIAN PORTUGUESE SPEAKERS)

**Table:**

Figure	Present Alteration	Figure	Present Alteration
Clock		Cockroach	
Pencil		Strawberry	
Cat		Giraffe	
Dice		Door	
Little Bird		Boat	
Sofa		Fork	
Scissors		Dish	
Home		Train	
Bicycle		Dragon	
Star		Book	
Truck		Plate	
Eye		Arrow	
Key		Blouse	
Airplane		Flute	
Butterfly		Bell	
Dog		Bone	
Telephone		Zebra	
Flower		Blue wing	
Gift		Umbrella	
Alligator		Hat	
Hammer		Window	
Cross		Ladybird	
Grass		Chicken	
Owl		Crown	
Athlete		Globe	

**Source:** Chart updated by the authors, based on the original version of the Tongue frenulum evaluation protocol^(22)^
