# Supplementary material for: Reduced version of the MBGR protocol: validity based on content and the response processes
Source: Codas. 2026 Jul 31;38(5):e20250355. doi: 10.1590/2317-1782/e20250355en (PMC13440979; doi:10.1590/2317-1782/e20250355en)
Supplement: Translation

# Translation: Versão reduzida do protocolo de exame miofuncional orofacial MBGR: evidência de validade baseada no conteúdo e nos processos de resposta

**DOI:** 10.1590/2317-1782/e20250355pt

**Authors:** Asenate Soares de Matos Pereira, Herick Santos Assis, Paola Pereira Leite, Katia Flores Genaro, Giédre Berretin-Felix

**Affiliations:** 1 Departamento de Fonoaudiologia, Faculdade de Odontologia de Bauru – FOB, Universidade de São Paulo – USP - Bauru (SP), Brasil.

**Keywords:** Fonoaudiologia, Protocolos Clínicos, Estudo de Validação, Avaliação em Saúde, Sistema Estomatognático

## Abstract

**Objetivo:**

Diante da necessidade clínica de agilidade avaliativa, pretendeu-se apresentar a versão reduzida do Protocolo de Exame Miofuncional Orofacial MBGR, com evidências de validade baseada no conteúdo e nos processos de resposta.

**Método:**

Uma versão prévia do Protocolo MBGR reduzido foi elaborada a partir da versão original e submetida à metodologia Delphi. A validação do conteúdo feita em duas análises, utilizou escala do tipo Likert para avaliar a relevância/representatividade dos itens, e calculou o Índice de Validade de Conteúdo (IVC). Onze fonoaudiólogos foram treinados e aplicaram o protocolo em 11 pacientes e, posteriormente, responderam a um questionário, cujas informações foram discutidas em comitê. O teste t pareado ou o teste de Wilcoxon foram usados para comparação entre as versões original e reduzida, considerando o nível de significância de 5%.

**Resultados:**

A proposta inicial do protocolo reduzido contemplou 36 itens. Na avaliação realizada pelo comitê constituído por 11 especialistas, sete analisaram e emitiram parecer sobre cada item, possibilitando a exclusão de 17 itens. A evidência de validade de conteúdo do protocolo apresentou IVC global de 87% e os fonoaudiólogos relataram adequada compreensão e aplicabilidade da versão reduzida, sendo que a maioria considerou o protocolo original extenso e a versão reduzida mais viável na prática clínica. As sugestões foram majoritariamente acatadas, resultando em ajustes e exclusões que finalizaram o protocolo reduzido, com escore de 0 a 137 pontos.

**Conclusão:**

Obteve-se a evidência de validade baseada no conteúdo e dos processos de resposta da versão reduzida do Protocolo de Exame Miofuncional Orofacial MBGR.

## INTRODUÇÃO

A área da Motricidade Orofacial (MO) é voltada para o diagnóstico das alterações miofuncionais orofaciais, realizado por meio do exame funcional e morfológico do sistema estomatognático (SE)^(1)^. Para que essa atuação seja menos propensa a erros^(1)^, fundamenta-se a utilização de protocolos validados, no intuito de direcionar o olhar clínico e acompanhar a evolução do caso ao longo do processo de tratamento^(2)^.

Dentre os instrumentos validados e existentes na MO, o Protocolo de Avaliação Miofuncional Orofacial MBGR^(3)^ compreende, na sua proposta, os aspectos gerais do SE e as funções orofaciais de respiração, mastigação, deglutição e fala. Este protocolo é composto por duas seções principais: História Clínica e Exame Miofuncional Orofacial, abrangendo avaliação dos aspectos morfofuncionais e das funções. Além disso, inclui uma documentação, que envolve fotografias e filmagens, para registro e análise posterior.

O Protocolo MBGR^(3)^ tem sido amplamente utilizado para avaliação e diagnóstico de alterações miofuncionais orofaciais em variadas populações, tais como indivíduos com Síndrome da Apneia Obstrutiva do Sono – SAOS^(4)^ e em estudos na área da estética^(5)^. Sabe-se também que foi ampliado e validado um Protocolo de Avaliação Miofuncional Orofacial para indivíduos com fissura labiopalatina, adaptado do protocolo MBGR^(6)^, como também foi validado para adultos com DTM^(7)^. Em relação à faixa etária, esse instrumento foi aplicado nos diferentes ciclos da vida, em crianças e adolescentes^(8,9)^, em adultos^(10,11)^, em idosos^(12)^ e, mais recentemente, foi adaptado para lactentes e pré-escolares^(13,14)^. Por outro lado, o protocolo é extenso e pode dificultar sua aplicação em situações que necessitem de uma avaliação mais rápida. Portanto, a redução do conteúdo do instrumento contribuirá para o avanço científico no processo de diagnóstico dos distúrbios miofuncionais orofaciais (DMO), com impacto para a prática clínica, facilitando a sua utilização.

Para propor a redução do Protocolo MBGR é necessário cumprir diretrizes internacionais que garantam as propriedades psicométricas^(15)^, seguindo a sistematização do percurso metodológico para concluir suas etapas de evidências de validade, já que essa padronização tem possibilitado resultados cada vez mais confiáveis^(1)^. Assim, diante da necessidade clínica de uma avaliação mais ágil e viável quanto ao tempo de aplicação, o objetivo deste artigo é apresentar a versão reduzida do Protocolo de Exame Miofuncional Orofacial MBGR, abordando as etapas de evidência de validade baseada no conteúdo e nos processos de resposta.

## MÉTODO

Trata-se de um estudo metodológico com abordagem descritiva e quantitativa, voltado ao desenvolvimento da versão reduzida do Protocolo de Avaliação Miofuncional Orofacial MBGR pela técnica Delphi^(16)^ adaptada, após obter-se parecer favorável à adaptação pelas autoras do protocolo MBGR original^(3)^.

### Aspectos éticos

Projeto aprovado pelo Comitê de Ética em Pesquisa (CEP) com Seres Humanos da instituição onde o estudo foi realizado (CAE: 61170822.3.0000.5417, parecer: 5.648.926). Participaram do estudo pacientes e fonoaudiólogos mediante a assinatura do Termo de Consentimento Livre e Esclarecido, e todos foram claramente informados sobre os objetivos da pesquisa, riscos e benefícios.

### Procedimentos

O estudo contemplou as seguintes etapas: 1) elaboração da versão preliminar reduzida do Protocolo de Exame Miofuncional Orofacial MBGR pelas autoras do protocolo original, 2) Evidência de validade baseada no conteúdo, 3) Evidência de validade baseada nos processos de resposta e 4) aprovação/adequação da versão final do protocolo reduzido pelas autoras do protocolo original.

1. Elaboração da versão preliminar reduzida do Protocolo de Exame Miofuncional Orofacial MBGR

Foi realizado o contato com as autoras do Protocolo MBGR original, para informar o objetivo da pesquisa e solicitar a participação delas na elaboração da versão reduzida. Em seguida, essas autoras analisaram, individualmente, o protocolo e propuseram a extinção ou modificação de itens/subitens. Após essa tarefa, as autoras se reuniram para discutir uma proposta da versão reduzida, sendo todas as sugestões registradas e as alterações definidas em consenso. Assim, a versão preliminar reduzida do Protocolo de Exame Miofuncional Orofacial MBGR foi concluída.

1. Evidência de validade baseada no conteúdo

Foram convidados 11 fonoaudiólogos, denominados examinadores, para realizar a análise da versão preliminar reduzida do protocolo, considerando como critérios: ter utilizado a versão original do protocolo durante a graduação em Fonoaudiologia, e também ter experiência na utilização desse protocolo em sua prática profissional.

Em relação ao perfil profissional dos fonoaudiólogos, o tempo de formação variou de 1 a 15 anos, 54,54% fizeram ou faziam residência, 63,63% eram mestres ou mestrandos e 45,45% eram doutores ou estavam cursando doutorado. Ressalta- se que 100% dos profissionais tiveram alguma experiência de aplicação com o MBGR na versão original, conforme critério de inclusão adotado.

A versão preliminar reduzida do Protocolo de Exame Miofuncional Orofacial MBGR foi inserida em uma plataforma Google Forms®, de forma que, para cada um dos itens/subitens, uma escala Likert com pontuação de um a quatro foi utilizada para analisar a sua relevância/representatividade: 1 = não relevante/não representativo, 2 = necessita grande revisão para ser relevante/representativo, 3 = necessita de pequena revisão para ser relevante/representativo, 4 = relevante/representativo^(17)^. Ao final de cada item havia um espaço para adicionar a justificativa da resposta, dar sugestões ou deixar algum comentário.

Após o aceite do convite pelos examinadores, foi encaminhado o link de acesso à plataforma do Google Forms®, para realizarem uma primeira análise. As respostas das análises foram tabuladas, os comentários e sugestões foram categorizados, e os ajustes necessários foram realizados, dando origem ao protocolo preliminar reduzido reformulado. Em seguida, foi feito contato novamente com os examinadores para esclarecimentos e devolutivas sobre a sua primeira análise, e convidando-os a realizarem uma segunda análise, agora da versão preliminar reduzida reformulada, da mesma forma como realizado na primeira análise. Novamente alguns ajustes foram necessários, a partir das sugestões.

A concordância entre os avaliadores foi analisada por meio do Índice de Validade de Conteúdo (IVC)^(18)^, com o cálculo realizado no Microsoft Excel 2019®. Primeiro, foi calculado o IVC de cada item individualmente, considerando-se a proporção de respostas com pontuação 3 ou 4 em relação ao total de avaliadores. Em seguida, obteve-se o IVC global do protocolo a partir da média dos valores de IVC dos itens avaliados em cada rodada. Valores iguais ou superiores a 0,80 foram considerados indicativos de adequação e representatividade dos itens.

Após tais análises, a versão preliminar reduzida e reformulada do protocolo retornou aos profissionais, com as mudanças solicitadas nos itens pontuados 1 e 2, para reanálise das modificações/adequações realizadas. As sugestões também foram analisadas para a versão pré-final.

1. Evidência de validade baseada nos processos de resposta

Este procedimento envolveu quatro fases e foi adaptado de estudos sobre padronização, treinamento de examinadores^(14)^, aplicabilidade e parecer documentados^(19)^ e garantia de que a versão simplificada atenda aos critérios do protocolo original^(20)^. Fase 1: treinamento dos fonoaudiólogos; fase 2: aplicação do protocolo reduzido; fase 3: sugestões imediatamente após a aplicação do protocolo e análise comparativa da versão reduzida com o protocolo original; fase 4: análise pelas autoras do protocolo original.

Para essa etapa, foram selecionados 11 pacientes de uma Clínica Escola de Fonoaudiologia, diagnosticados com distúrbio miofuncional orofacial, com idade entre 19 a 44 anos. Foi feito contato telefônico com eles para explicar o objetivo da pesquisa, como seria a sua participação e verificar a disponibilidade de horário.

Destaca-se que o número de participantes foi definido para corresponder ao número de avaliadores da pesquisa, que seguiu recomendações metodológicas para a etapa de evidência de validade baseada nos processos de resposta^(15, 19)^. Os critérios de inclusão foram: idade compatível com a aplicação do protocolo (crianças, adolescentes ou adultos), possibilidade de aplicação completa da versão reduzida do MBGR e identificação prévia de alteração miofuncional orofacial. Todos os participantes contactados atenderam aos critérios estabelecidos, concordaram em participar mediante assinatura do TCLE e concluíram integralmente a aplicação, não havendo desistências ou perdas amostrais.

Na fase 1 foi realizado um treinamento dos examinadores com mensuração do tempo necessário para orientações, com base no manual de utilização do MBGR^(21)^, sendo explicado o objetivo do protocolo, o conceito de cada questão e como seria feita a aplicação da versão reduzida. Na fase 2, a aplicação do protocolo reduzido foi realizada presencialmente, sendo o atendimento registrado em vídeo (câmera Intelbras, modelo iM5), com o propósito de mensurar o tempo de aplicação do protocolo.

Na fase 3, os examinadores receberam um questionário composto por 17 perguntas sobre a versão reduzida do protocolo, a ser respondido imediatamente após a aplicação desse protocolo. Havia perguntas relacionadas à compreensão e aplicação do protocolo reduzido (1, 3, 5, 7, 9, 11 e 13), sendo utilizado uma escala tipo Likert na análise: 1 = concordo totalmente; 2 = concordo; 3 = nem concordo, nem discordo; 4 = discordo parcialmente e 5 = discordo totalmente. Haviam, ainda, perguntas que se relacionavam à relevância/importância dos itens (2, 4, 6, 8, 10, 12 e 14), também analisadas por meio de uma escala Likert: 1 = muito importante; 2 = importante; 3 = mediano; 4 = às vezes é importante e 5 = não é nada importante. Por fim, a pergunta 15 solicitava que os examinadores apontassem se consideraram o protocolo extenso, a pergunta 16 questionava se o protocolo era aplicável na prática clínica, enquanto na 17 havia espaço para sugestões e comentários.

Transcorridos 8 dias, os examinadores responderam, de forma online, ao mesmo questionário, agora comparando as versões original e reduzida Protocolo MBGR. As questões foram realizadas, visando verificar a compreensão e a importância dos itens, bem como obter sugestão de mudança, sendo analisado todo o protocolo reduzido. As respostas foram tabuladas e submetidas à análise qualitativa, por meio da descrição das respostas, e à análise quantitativa, com comparação entre as versões original e reduzida do protocolo. Inicialmente, a distribuição dos dados foi avaliada quanto à normalidade. Para variáveis com distribuição normal, utilizou-se o teste t pareado; quando a normalidade não foi atendida, empregou-se o teste de Wilcoxon para amostras pareadas. Adotou-se nível de significância de 5%.

Na Fase 4, duas das autoras do protocolo original analisaram a versão final do protocolo reduzido, tendo sido necessário realizar pequenas adequações, identificadas a partir de suas experiências no ensino, pesquisa e na prática clínica. Nesse processo, também se decidiu pela atualização das *Pranchas de figuras* e do *Quadro para anotação da avaliação da Fala,* contidas no artigo *“Protocolo de avaliação do frênulo da língua”* ^(22)^, mediante autorização prévia da revista. As novas ilustrações foram desenvolvidas pelos autores com o auxílio da ferramenta de Inteligência Artificial ChatGPT (modelo GPT-5, OpenAI), preservando a proposta original e assegurando a padronização das imagens. Esse material poderá ser usado durante a aplicação da versão reduzida do Protocolo MBGR, de modo específico na avaliação do item “Funções Orofaciais”, para o subitem “Fala”.

## RESULTADOS

1. Elaboração da versão preliminar reduzida do Protocolo MBGR

O Protocolo MBGR original continha 10 páginas e 8 itens e passou para 5 páginas e 6 itens na versão reduzida. Em relação à pontuação geral, o valor máximo que indicava pior condição, era 353 e passou para 137. Para a versão reduzida, alguns itens ou subitens foram excluídos.

O escore final pode variar de 0 a 137 pontos, sendo que menores pontuações refletem melhor equilíbrio miofuncional orofacial. Dessa forma, a interpretação clínica deve considerar o valor obtido associado à análise do examinador.

O item 1. Postura Corporal foi retirado por não trazer dados significativos para a versão reduzida. No item 2. Medidas da Face, dos Movimentos Mandibulares e da Oclusão os subitens foram unificados e retiradas medidas consideradas “extras” para uma avaliação diminuta, finalizando com 9 medidas. Foram mantidas as 3 medidas com o paquímetro, para manter a veracidade dos resultados numa avaliação breve.

Já o item 3. Exame Extraoral, em relação à face foram retirados os subitens Tipo Facial e Análise Facial Subjetiva, pois tais análises necessitam apenas a observação do avaliador, e mantidos Proporção Facial, Simetria Facial e Padrão Facial. Para os Lábios, os subitens foram mantidos, com alteração na opção de resposta para a Forma do Inferior: três opções mais gerais para manter a eficiência. O subitem Masseter foi excluído, por se tratar de avaliação com menor importância dentre os outros mantidos.

No item 4. Exame Intraoral foram mantidos os seus subitens com algumas modificações: opção de resposta adequada ou alterada para Lábios e Bochechas; excluída a análise da Simetria e Altura da Língua; assim como a Coloração para as Tonsilas Palatinas e Linha Média, Guia de Desoclusão e Uso de Aparelho na Oclusão.

No item 5. Mobilidade foram mantidos os seus subitens, mas retiradas algumas possibilidades de resposta, sendo mantidas somente 3 opções. O item 6. Sensibilidade foi excluído, pois para uma avaliação breve não há benefício ter um item muito específico para o diagnóstico. Já para o item 7. Tônus, foram mantidas as avaliações de forma geral.

No item 8. Funções Orofaciais, para a Respiração foi retirado o subitem Tipo e modificado o Fluxo Nasal; para a Mastigação foram retirados: Números de ciclos, Mastigação ruidosa, Contrações musculares não esperadas e Perguntar ao paciente; na Deglutição foi mantida apenas a Deglutição habitual de líquido; para a Fala, o Aspecto Fonético/Fonológico foi modificado, permanecendo: Omissão, Substituição, Distorção, Projeção de Língua e Troca de Ponto Articulatório, com opção de resposta Ausente ou Presente, excluídos os subitens Prova Terapêutica, Função Velofaríngea e Voz, sendo este último adicionado ao subitem Aspectos Gerais e, especificamente para os Aspectos Gerais, foram retirados os subitens Movimentos de Lábios e da Língua, Posição de Língua na Fala e Entonação; por fim, o subitem Coordenação Motora na Fala foi reduzido para apenas avaliar a velocidade e o ritmo de “PATAKA”.

1. Evidência de validade baseada no conteúdo

Após a primeira análise pelos examinadores, os itens que receberam pontuação 4 ou 5, por pelo menos dois examinadores, foram alterados. Tais itens foram: Medidas da Face, dos Movimentos Mandibulares e da Oclusão; Exame Extraoral (Face); Exame intraoral (Lábios, Bochechas, Palato, Tonsila e Oclusão); Mobilidade (Mandíbula); Funções Orofaciais (respiração, aspectos gerais e coordenação motora na fala). Os demais itens foram mantidos, pois receberam pontuação 1 e 2. Ao calcular o IVC verificou-se resultado satisfatório (Tabela 1).

**Tabela 1:** Resultado do Índice de Validade de Conteúdo para a versão reduzida do Protocolo MBGR

**Item**		**IVC 1ª análise**	**IVC 2ª análise**
**1 - Medidas da Face, dos Movimento Mandibulares e da Oclusão**		0,85	1
	Face	0,71	1
**2 - Exame extraoral**			1
	Lábios	1	
	Lábios	0,85	1
	Bochechas	0,71	*
	Língua	1	1
**3 - Exame intraoral**	Palato	0,85	0,85
	Tonsila	0,85	1
	Dentes	1	1
	Oclusão	0,85	1
	Lábios	1	1
	Língua	1	0,85
**4 - Mobilidade**			
	Véu palatino	1	1
	Mandíbula	0,71	0,85
**5 - Tônus**		1	1
	Respiração	0,85	0,85
	Mastigação	1	1
	Deglutição	1	1
**6 - Funções Orofaciais**			
	Fala	1	1
	Aspectos gerais	0,71	1
	Coordenação motora na fala	0,71	^*^
**TOTAL**		18,65 / 21 = **0,88**	16,55/19 = **0,87**

**Legenda:** IVC = Índice de Validade de Conteúdo;

* item excluído a partir das sugestões dos avaliadores

Na segunda análise, dos 19 itens, 15 deles receberam pontuação 1 e 2 e apenas quatro receberam pontuação 4, sendo Exame Intraoral (Palato), Mobilidade (Língua e Mandíbula) e Funções Orofaciais (Respiração). Dentre os 15 itens, seis receberam de todos os examinadores a pontuação 1, sendo: Exame Intraoral (Lábios, Língua), Mobilidade (Véu Palatino), Funções orofaciais (Mastigação, Fala e Aspectos Gerais), enquanto oito itens receberam pontuações entre 2 e 3, sendo: Medidas da Face, dos Movimentos Mandibulares e da Oclusão, Exame Extraoral (Face), Exame Intraoral (Lábios, Tonsilas Palatinas, Dentes e Oclusão), Mobilidade (Lábios), Tônus e Funções Orofaciais (Deglutição). Dos 19 itens, 15 apresentaram concordância perfeita, com IVC=1 (Tabela 1).

O resultado global e final do IVC para o protocolo como um todo foi de 0,87 (87%), acima do valor mínimo considerado aceitável.

1. Evidência de validade baseada nos processos de resposta

Os resultados da fase 1 mostraram tempo mínimo de 20 e máximo de 62 minutos, com uma mediana de 56 minutos para treinamento dos examinadores, com média de 51,72 minutos e desvio-padrão de ±12,37. Quanto ao tempo para aplicação do protocolo reduzido (fase 2), foi verificado mínimo de 34 e máximo 64 minutos, com mediana de 40 minutos, média de 42,45 minutos e desvio-padrão de ±11,83, evidenciando maior eficiência clínica na aplicação do Protocolo MBGR reduzido.

Para a fase 3, no que se refere à compreensão e aplicação (perguntas 1, 3, 5, 7, 9 e 11), a comparação da pontuação atribuída pelos examinadores para ambas as versões (reduzida e original) do Protocolo MBGR, por meio dos testes de Wilcoxon e t pareado, não mostrou diferença (Tabela 2). Esse resultado indica que os examinadores conseguiram compreender e aplicar os itens: Medidas da face, dos movimentos mandibulares e da oclusão; Exame extraoral, Exame intraoral; Mobilidade; Tônus e Funções Orofaciais em ambos os protocolos.

**Tabela 2:** Análise das respostas dos examinadores sobre compreensão e aplicação das versões reduzida e original do Protocolo MBGR para os itens Medidas da face, dos movimentos mandibulares e da oclusão; Exame extraoral, Exame intraoral; Mobilidade; Tônus e Funções Orofaciais

	**n**	**Mediana**	**25%**	**75%**
P1 - Reduzido	11	1,000	1,000	1,000
P1 - Original	11	1,000	1,000	2,000
P3 - Reduzido	11	1,000	1,000	2,000
P3 - Original	11	1,000	1,000	2,000
P5 - Reduzido	11	1,000	1,000	1,000
P5 - Original	11	1,000	1,000	2,000
P9 - Reduzido	11	1,000	1,000	1,000
P9 - Original	11	1,000	1,000	2,000
P11 - Reduzido	11	1,000	1,000	2,000
P11 - Original	11	1,000	1,000	2,000
	**n**	**Média**	**Desvio Padrão**	**SEM**
P7 - Reduzido	11	1,273	0,467	0,141
P7 - Original	11	1,455	0,934	0,282
Diferença		-0,182	0,874	0,263

**Legenda:** n = número de examinadores; SEM = *Structural Equation Modeling*

Já, quanto à relevância/importância (perguntas 2, 4, 6, 8, 10, 12 e 14), obteve-se resultados distintos. Na pergunta 2 (medidas da face, dos movimentos mandibulares e da oclusão), para a versão reduzida, 64% dos examinadores avaliaram esse item como muito importante e, para a versão original, houve variação quanto a avaliação: 55% classificaram como muito importante ou importante, 18% como mediano e 27% como às vezes é importante. Já, na pergunta 12 (Funções Orofaciais), 91% dos examinadores avaliaram este item como muito importante ou importante em ambas as versões, enquanto 9% avaliaram como mediano na versão reduzida e 9% como às vezes importante na versão original. A aplicação do teste de Wilcoxon não mostrou diferença, indicando que os examinadores classificam os itens Medidas da face, dos movimentos mandibulares e da oclusão, bem como Funções Orofaciais importantes para ambos os protocolos (Tabela 3).

**Tabela 3:** Análise das respostas dos examinadores sobre a relevância/importância dos itens Medidas da face, dos movimentos mandibulares e da oclusão; Exame extraoral, Exame intraoral; Mobilidade; Tônus e Funções Orofaciais para as versões reduzida e original do Protocolo MBGR

	**n**	**Mediana**	**25%**	**75%**	
P2 - Reduzido	11	1,000	1,000	3,000	
P2 - Original	11	1,000	1,000	4,000	
P12 - Reduzido	11	1,000	1,000	1,000	
P12 - Original	11	1,000	1,000	1,000	
	**n**	**Média**	**Desvio Padrão**	**SEM**	**Valor de p**
P4 - Reduzido	11	1,545	0,522	0,157	
P4 - Original	11	3,000	1,000	0,302	**p< 0,001**
Diferença		-1,455	0,934	0,282	
P6 - Reduzido	11	1,273	0,467	0,141	
P6 - Original	11	2,636	1,690	0,509	**p< 0,001**
Diferença		-1,364	1,804	0,544	
P8 - Reduzido	11	1,636	1,027	0,31	
P8 - Original	11	2,000	1,095	0,33	p > 0,05
Diferença		-0,364	0,924	0,279	
P10 - Reduzido	11	1,545	0,934	0,282	
P10 - Original	11	1,636	0,809	0,244	p > 0,05
Diferença		-0,0909	0,944	0,285	

**Legenda:** n = número de examinadores; SEM = *Structural Equation Modeling*

Nas perguntas 4 (Exame extraoral) e 6 (Exame intraoral), a análise pelo teste t pareado mostrou que a média da pontuação atribuída pelos examinadores à versão reduzida foi menor (p<0,001), demonstrando ser itens com relevância/importância; diferentemente do resultado das perguntas 8 (Mobilidade) e 10 (Tônus), em que o teste t pareado não evidenciou diferença entre as médias das pontuações das versões original e reduzida (Tabela 3).

Em relação à folha de resumo do protocolo e a sugestão de roteiro para registro de imagens (perguntas 13 e 14), a maioria dos examinadores sugeriu a sua exclusão, justificada pelo impacto no tempo de aplicação e ausência de valores de referência para comparação. Ainda assim, optou-se por manter o resumo e o roteiro, visando uma futura validação com a definição desses parâmetros.

Os resultados da pergunta 15 sobre a extensão dos protocolos mostrou para a versão reduzida que 73% discordaram total (36,5%) ou parcialmente (36,5%) que essa seja extensa, 9% não concordaram nem discordaram e 18% a consideraram extensa; quanto à versão original, 73% concordaram total (55,5%) ou parcialmente (36,5%) e 9% discordaram que seja extensa. Tais resultados demonstraram, pelo teste de Wilcoxon, que a versão reduzida não é extensa comparada à versão original (Tabela 4).

**Tabela 4:** Análise das respostas dos examinadores sobre extensão e aplicabilidade de ambas as versões do protocolo MBGR

	**n**	**Mediana**	**25%**	**75%**
P15 - Reduzido	11	2,000	1,000	3,000
P15 - Original	11	5,000	4,000	5,000
P16 - Reduzido	11	2,000	1,000	2,000
P16 - Original	11	4,000	3,000	4,000

**Legenda:** n = número de examinadores

Na questão 16 foi questionado os examinadores sobre a aplicabilidade da versão reduzida na prática clínica, sendo que 91% concordaram total (45,5%) ou parcialmente (45,5%) e 9% discordou; em relação à versão original, 18% concordaram, 27% nem concordaram nem discordaram, 55% discordaram parcial (45,5%) ou totalmente (9,5%). Sendo assim, esses resultados mostram que a maioria dos examinadores concordaram total ou parcialmente ser a versão reduzida aplicável na prática clínica, enquanto 55% discordaram total ou parcialmente ser versão original seja aplicável na prática clínica, sendo essa diferença observada no teste de Wilcoxon (Tabela 4).

Outra análise realizada foi a comparação quanto a compreensão, aplicação e importância de cada item, após 8 dias da aplicação da versão reduzida, sendo obtidos comentários positivos dos examinadores, reafirmando a importância dos itens da versão reduzida (Quadro 1), como sugestões (Quadro 2).

**Quadro 1:** Comentários positivos dos examinadores sobre a versão reduzida

**MEDIDAS DA FACE, DOS MOVIMENTOS MANDIBULARES E DA OCLUSÃO**	
Compreensão e aplicação	1. Importante para entender as proporções faciais e compreender as limitações estruturais.
	2. As explicações sobre cada item são importantes para relembrar em caso de esquecimento.
	3. As instruções são claras e compreensíveis.
Importância	4. Esse item indica o que podemos esperar nos próximos itens de avaliação.
	5. As medidas de movimento mandibular e oclusão colaboram para o diagnóstico fonoaudiológico.
**EXAME EXTRAORAL**	
Compreensão e aplicação	1. Ajuda a investigar características que indicam alteração estrutural e funcional.
	2. As instruções são claras e compreensíveis.
Importância	3. Padrão facial, postura dos lábios e mucosa colaboram para o diagnóstico fonoaudiológico.
	4. Importante para corroborar ou não com o comentário do paciente sobre a sua respiração.
	5. É importante e dá indícios da tonicidade das estruturas.
	6. As condições extraorais podem ter influência sobre as funções e, portanto, considero importante avaliá-las mesmo em uma versão reduzida.
**EXAME INTRAORAL**	
Compreensão e aplicação	1. Todos os itens estão claros.
	2. As instruções são claras e compreensíveis.
	3. Avalia as características intraorais e facilita a compreensão de funcionalidade.
	4. Importante, principalmente para saber a saúde bucal do paciente, até para indicar e procurar profissionais em caso de suspeita de alterações, como câncer ou má higienização.
	5. Esse item é essencial.
**MOBILIDADE**	
Compreensão e aplicação	1. Compreendo/concordo com a presença dos quatro itens de mobilidade.
	2. As instruções são claras e compreensíveis.
	3. Item indispensável.
**TÔNUS**	
Compreensão e aplicação	1. As instruções são claras e compreensíveis.
Importância	2. É possível relacionar com os outros itens presentes, como exame extraoral, mastigação, mobilidade.
	3. Acredito que sim, em caso de alterações as funções podem ser prejudicadas.
**FUNÇÕES OROFACIAIS**	
Compreensão e aplicação	1. As instruções são claras e compreensíveis.
Importância e avaliação	2. Após avaliar as estruturas é necessário saber como elas estão funcionando, então é extremamente importante.
	3. Indispensável.

**Quadro 2:** Sugestões dos examinadores sobre a versão reduzida

**MEDIDAS DA FACE, DOS MOVIMENTOS MANDIBULARES E DA OCLUSÃO**		**MA**
Compreensão e aplicação	1. Qual seria o valor a se considerar no caso de discrepância (deixar escrito).	-
Importância	2. Pensando na versão reduzida, talvez realizar apenas uma medida.	SIM
	3. As medidas de movimento mandibular e oclusão colaboram para o diagnóstico fonoaudiológico, mas medidas da face não.	NÃO
	4. Seria interessante uma pequena imagem de TV e TH ao lado, ou em substituição da descrição para ajudar a lembrar o profissional a medir.	SIM
	5. Movimentos mandibulares são importantes, medidas da face seria melhor em casos específicos.	NÃO
	6. Acredito que para a prática clínica esse item poderia ser suprimido.	NÃO
**EXAME EXTRAORAL**		
Compreensão e aplicação	1. Seria interessante discriminar que a proporção facial é obtida a partir dos dados das medidas da face, semelhante as descrições anteriores. Da mesma forma, côncavo e convexo, para mim, são termos difíceis, que não auxiliam muito.	SIM
Importância	2. Pode ser melhor aproveitado em casos específicos e não de modo geral.	NÃO
**EXAME INTRAORAL**		
Importância	1. Tiraria apenas o tópico "**Nº de dentes**".	SIM
	2. O exame intraoral pode ser reduzido, por exemplo: "cavidade oral": onde se pontue os aspectos alterados encontrados de forma geral, apenas. Sem citar itens adequados, mas observando todos.	NAO
	3. Acredito que a **contagem de dentes** não é necessária na versão reduzida.	SIM
**MOBILIDADE**		
Compreensão e aplicação	1. Corrigir apenas a letra E de lado esquerdo.	SIM
Importância	2. Torna-se secundário, mediante alterações das funções. Se a função estiver alterada, aí vale a pena investigar.	NÃO
	3. Acredito que alternância dos lábios não é importante, como bem como, **mobilidade do véu palatino e mandíbula**. A menos que o paciente apresente queixa ou quadro relacionado a isso, podendo ser colocado em "observação".	NÃO
	4. Acredito que o item **do véu palatino (e mandíbula)** não são necessários já que é uma versão reduzida.	NÃO
**TÔNUS**		
	Não houve sugestões.	
**FUNÇÕES OROFACIAIS**		
Compreensão e aplicação	1. Na parte da possibilidade de uso nasal, indicar que é para cronometrar ou por escrito ou com o símbolo de um relógio.	SIM
Importância	2. O paciente apresentou alterações na deglutição de sólido que não foram anotadas por não ter um campo específico.	SIM
	3. Na fala deixaria o tópico amostra **de fala espontânea**.	SIM
	4. São os aspectos mais importantes a serem avaliados. Seria mais rico, inclusive, uma maior amostra para avaliação de fala (contagem de dias, da semana, **fala espontânea**).	SIM
	5. Por ser uma versão reduzida, acredito que não seria tão importante os itens de possibilidade de uso nasal, verificando apenas o fluxo nasal. E sobre a mastigação tudo OK.	NÃO

**Legenda:** MA = Modificação Atendida

Analisando os comentários dos examinadores, verificou-se que alguns sugeriram mudanças na versão reduzida, sendo as sugestões não acatadas devidamente justificadas. Das modificações realizadas na versão reduzida, entende-se que as sugestões foram necessárias para aprimorar a qualidade do protocolo.

Em relação ao item Medidas da face, dos movimentos mandibulares e da oclusão, a primeira sugestão não foi acatada, pois a segunda foi. Foram então excluídas a 2ª e 3ª medidas, sem necessidade de registrar o valor no caso de discrepância. A terceira sugestão de alteração não foi possível realizar, tendo em vista que as medidas da face também colaboram para o diagnóstico fonoaudiológico. Por sua vez, a quarta sugestão foi acatada e aplicada para outras medidas, sendo adicionadas imagens para facilitar e relembrar a execução das medidas. A última sugestão não foi considerada, já que este item não pode ser excluído na íntegra, por ser esta análise de grande importância na avaliação estrutural.

No exame extraoral, a primeira sugestão foi considerada, visando a facilidade em aplicar a versão reduzida em um menor tempo, mas a segunda não foi considerada, pois o exame extraoral faz parte da avaliação estrutural.

Em relação ao exame intraoral, dois profissionais diferentes fizeram a sugestão de excluir a contagem de dentes, o que foi acatado; foram adicionadas imagens das dentições decídua e permanente para ilustrar. Por outro lado, a sugestão de uma avaliação de forma geral, sem citar o item analisado, não foi acatada, pois o protocolo tem como objetivo a padronização da avaliação.

Quanto à mobilidade, a sugestão de correção da letra E para representar o lado esquerdo foi considerada e as demais não puderam ser adotadas. O item tônus não recebeu sugestão.

Para as funções orofaciais (respiração, mastigação, deglutição e fala) foram analisadas cinco sugestões. A primeira foi acatada e se referiu a inserir o símbolo de um relógio para indicar a necessidade de cronometrar a “possibilidade de uso nasal”, sendo incluído também o símbolo de um copo com líquido, indicando a necessidade de manter um volume de água na boca durante a prova, visando a praticidade na aplicação da versão reduzida do protocolo em uma menor quantidade de tempo. Como foi observado que o profissional sugeriu imagens para facilitar a aplicação, funcionando como um lembrete, essa sugestão foi recebida e ampliada para outras seções no protocolo, que poderiam ser adicionadas imagens, com objetivo de facilitar a aplicação. A segunda sugestão, para adicionar um campo específico, não foi acatada, pois os dados extras podem ser anotados no campo “observação”. As terceira e quarta sugestões, para manter na avaliação da fala as amostras de fala espontânea e automática, foram consideradas, sendo inclusive, adicionadas ao protocolo a informação de que na avaliação de fala, o fonoaudiólogo pode utilizar os dois aspectos. Por último, a quinta sugestão, para exclusão da possibilidade de uso nasal, não foi acatada, tendo em vista que tal aspecto representa um item importante para direcionar a terapia de pacientes com respiração oronasal.

Na Fase 4, a versão final do protocolo reduzido foi apresentada às autoras do protocolo original, que consideraram pertinente a maioria dos ajustes. No item Medidas da face, dos movimentos mandibulares e da oclusão, as palavras "terço" e "lábios", grafadas com inicial minúscula, foram corrigidas para inicial maiúscula; também foram incluídas as siglas: LS (Lábio Superior) e LI (Lábio Inferior). No item Exame extraoral, o termo "comprimento do superior" foi excluído e substituído por dois outros: "exposição dos incisivos superiores em repouso" e "proporção LS/LI".

No item Funções Orofaciais, foram inseridos para a respiração os aspectos "adequado", "reduzido unilateral" e "reduzido bilateral" no subitem "fluxo nasal".

Para a mastigação foi adicionado o subitem "eficiência", incluindo também os aspectos "bilateral alternado" e "unilateral alternado". Na deglutição, que anteriormente se restringia à avaliação de líquidos, o escopo foi ampliado para abranger também sólidos, sendo reformulado como "deglutição habitual de sólido e líquido". Consequentemente, o subitem "contenção do líquido", foi substituído por "contenção do alimento". Ademais, novos itens foram incluídos, abrangendo a avaliação dos movimentos de cabeça e da contração dos músculos orbicular da boca e mentual.

Quanto à fala, foi realizada exclusão do subitem “troca do ponto articulatório”, bem como substituição do subitem “projeção da língua” por “interdentalização lingual”.

Quanto as *Pranchas de figuras*, decidiu-se pela substituição da figura de um telefone antigo pela de um telefone celular, visando atualizar a imagem devido as mudanças tecnológicas e garantir maior reconhecimento pelas crianças, que apresentam dificuldade em identificar o modelo telefônico anterior. Em relação ao *Quadro para anotação da avaliação da Fala*, o termo ‘produção paciente’ foi substituído por ‘alteração presente’, com o objetivo de alinhar a terminologia ao aspecto fonético esperado na área da MO. Por fim, as 50 palavras da lista original foram mantidas^(22)^.

A partir das modificações realizadas, obteve-se a versão final do Protocolo MBGR Reduzido (MBGR-r), visualizado no Apêndice A.

## DISCUSSÃO

O desenvolvimento da versão reduzida do Protocolo de Avaliação Miofuncional Orofacial MBGR seguiu etapas de evidência de validade. Destaca-se como inovação deste estudo o uso de Inteligência Artificial (ChatGPT, modelo GPT-5, OpenAI) na atualização e padronização das ilustrações da versão reduzida do Protocolo MBGR. Essa abordagem contribuiu para maior uniformidade visual dos estímulos e representa uma interface emergente entre tecnologia e saúde, ampliando possibilidades para o desenvolvimento e modernização de instrumentos clínicos na área da Fonoaudiologia.

No processo de atualização das ilustrações, buscou-se preservar integralmente a estrutura, a organização e os referenciais das figuras originais, assegurando coerência com sua proposta clínica. As modificações realizadas tiveram caráter essencialmente gráfico, com o objetivo de padronizar os estímulos visuais e reduzir elementos que pudessem induzir identificação etária, geracional ou pessoal, tornando as imagens mais neutras e atemporais, principalmente quanto a figura “Telefone”. Dessa forma, a atualização não implicou substituição conceitual das figuras, mas sim adequação visual alinhada às transformações tecnológicas e comunicacionais.

Para um uso padronizado do protocolo, é fundamental aderir aos procedimentos de validação, respeitando as diretrizes que asseguram suas propriedades psicométricas, conforme recomendado pela literatura^(15)^. Os resultados do IVC indicaram que o protocolo reduzido possui boa representatividade dos itens^(21)^ essenciais para a avaliação miofuncional orofacial, com ajustes pontuais, sendo necessários para otimizar a aplicabilidade e a clareza dos itens. A análise contínua e a validação subsequente do protocolo, com a implementação dessas modificações, são passos importantes para garantir que o instrumento atenda às necessidades dos profissionais da área e proporcione diagnósticos mais precisos e eficientes^(16)^.

O resultado global de 87% do IVC indica que o Protocolo MBGR reduzido obteve evidência inicial de validade para uso clínico^(21)^, além de evidenciar que o processo de revisão foi conduzido de maneira rigorosa e colaborativa, com a integração de especialistas e ajustes baseados em evidências^(14,19,20)^. Esses resultados sugerem que o protocolo pode ser uma ferramenta eficaz para a avaliação, atendendo às necessidades de praticidade sem comprometer a qualidade diagnóstica. A partir dos resultados, pode-se inferir que o Protocolo MBGR reduzido apresenta uma maior aplicabilidade na prática clínica diária dos fonoaudiólogos em comparação com a versão original do protocolo. A redução no tempo de aplicação torna a versão simplificada mais eficiente, facilitando sua administração e tornando-a mais prática para uso em ambientes clínicos de rotina^(23)^. A literatura destaca a importância dessa tendência, já que o uso de protocolos extensos pode ser limitado em serviços de saúde devido à sua complexidade e à carga de respostas, o que torna essencial a adoção de versões mais curtas e objetivas^(24)^.

A não exclusão completa da seção de medidas da face, dos movimentos mandibulares e da oclusão reafirma a necessidade de uma avaliação estrutural detalhada, permitindo uma abordagem mais precisa e individualizada para cada paciente^(25)^. A manutenção dos valores normativos e esperados é fundamental para a detecção de desvios na normalidade^(26)^, pois podem estar associadas a problemas esqueléticos, funcionais e neuromusculares^(25)^ e, portanto, para que haja compreensão entre a relação do equilíbrio muscular e das estruturas orofaciais com as funções estomatognáticas^(3)^. A inclusão de imagens para ilustrar as medidas da face, dos movimentos mandibulares e da oclusão reflete uma preocupação com a padronização e reprodutibilidade do protocolo, uma vez que a literatura indica que o uso de recursos visuais contribui para minimizar erros de aferição e melhora a compreensão dos avaliadores, impactando positivamente na confiabilidade dos resultados^(27)^.

A não exclusão completa da seção de exame extraoral reforça a importância da avaliação de estruturas como lábios e face, ao analisar aspectos avaliados como o padrão facial, que auxilia na investigação nos casos de pacientes com alterações craniofaciais que podem predizer o risco de Distúrbio Miofuncional Orofacial^(26)^ e com má oclusão, classe II e classe III de Angle e seus componentes dentários e esqueléticos^(25)^.

No que se refere ao exame intraoral, a não aceitação da sugestão de uma avaliação intraoral mais generalista reforça a importância da padronização do protocolo. Entende-se que a avaliação padronizada permite maior confiabilidade na coleta de dados e evita interpretações subjetivas que poderiam comprometer a precisão diagnóstica^(2)^. A literatura sugere que avaliações detalhadas favorecem a identificação precoce de alterações estruturais e funcionais, essenciais para a prática fonoaudiológica, destacando a interação entre forma e função dos órgãos fonoarticulatórios, que, ao desempenharem suas funções motoras e neuromusculares, garantem a harmonia do complexo craniofacial^(28)^.

A seção de mobilidade não levou em consideração os dados sobre sugestões de exclusão. A análise das provas de mobilidade de lábios, língua e mandíbula permite a identificação das compensações musculares não envolvidas no movimento solicitado e um exemplo é a contração do músculo platisma durante a lateralidade mandibular, como uma compensação nos casos de disfunção muscular e articular^(25)^. A avaliação da mobilidade é crucial para observar a atividade muscular, diretamente relacionada às funções orofaciais e ao crescimento craniofacial^(2)^. A mobilidade do véu palatino é fundamental para detectar possíveis dificuldades respiratórias durante o sono^(4)^, e de modo específico, limitação nos movimentos da mandíbula também é importante em casos de radioterapia no local, no caso, de pacientes oncológico, e podem causar dificuldades no desempenho das funções orais^(29)^.

As modificações nas seções das funções orofaciais buscam equilibrar a praticidade e a manutenção de aspectos essenciais para a avaliação fonoaudiológica^(1)^. As sugestões acatadas, como a otimização e padronização do protocolo, estão alinhadas com a literatura^(23)^. Já as sugestões não implementadas, foram preservadas por sua importância no diagnóstico e direcionamento terapêutico.

A aprovação do protocolo final pelas autoras do Protocolo MBGR original valida a qualidade do trabalho, evidenciando que as modificações respeitaram os princípios e objetivos da avaliação fonoaudiológica. Todavia, uma limitação do estudo é a faixa etária dos pacientes avaliados, pois os mesmos tinham idades de 19 a 44 anos, tendo em vista que o MBGR original foi proposto para ser utilizado a partir de 6 anos. Além disso, embora o número de avaliadores esteja em consonância com recomendações metodológicas para estudos de validação, essa característica pode ser também considerada uma limitação do estudo, uma vez que um número maior de fonoaudiólogos poderia ampliar a diversidade de percepções e experiências clínicas.

Destaca-se ainda que a experiência prévia dos avaliadores com a versão original pode ter favorecido a aplicabilidade observada, já que a familiaridade com a estrutura e os critérios do protocolo tende a facilitar sua utilização, limitando a identificação de dificuldades que poderiam emergir em contextos com profissionais sem experiência prévia. Por fim, entende-se que os resultados obtidos indicam a necessidade e relevância de dar continuidade às demais etapas de validação da versão reduzida, seguindo o percurso metodológico previsto, a fim de ampliar evidências psicométricas e consolidar seu uso em diferentes contextos clínicos.

Considerando sua estrutura mais concisa e aplicabilidade facilitada, a versão reduzida do MBGR poderá apresentar potencial para uso em contextos de triagem ou teleavaliação, especialmente em cenários que demandem avaliações em menor tempo. Embora tais aplicações ainda exijam investigação específica, essa possibilidade poderá ampliar o alcance clínico e o desempenho do instrumento em diferentes contextos reais de atuação fonoaudiológica.

Em síntese, os resultados evidenciam a viabilidade e a pertinência da versão reduzida do Protocolo MBGR, contribuindo para uma atuação fonoaudiológica avaliativa mais ágil, objetiva e padronizada. Este estudo fortalece o campo da MO ao oferecer um instrumento com evidência inicial de validade e funcional, alinhado às demandas clínicas e à prática baseada em evidências. Estudos futuros poderão aprofundar análises psicométricas, estabelecer parâmetros normativos e ampliar seu uso em diferentes contextos, consolidando seu potencial como ferramenta de referência na área.

## CONCLUSÃO

Foi realizada a redução do Protocolo MBGR, de 36 itens para 19 itens no total, com pior pontuação de 353 para 134, respectivamente. Na avaliação global, o protocolo reduzido obteve o IVC de 0,87, superior ao valor aceitável. As sugestões dos examinadores foram consideradas, itens foram removidos, proporcionando e otimizando o uso do Protocolo MBGR reduzido. Este trabalho permitiu obter a evidência de validade baseada no conteúdo e dos processos de resposta, fornecendo a versão final do protocolo, com um menor tempo de aplicação.

Embora a etapa clínica, composta por 11 pacientes, ofereça evidência de aplicabilidade, seu tamanho reduzido limita a generalização dos achados. Assim, recomenda-se a continuidade das etapas de validação, incluindo análises de validade de construto, validade de critério e acurácia diagnóstica, a fim de ampliar a robustez psicométrica e consolidar o uso do protocolo em diferentes contextos clínicos, no diagnóstico dos distúrbios miofuncionais orofaciais.

## Figures and Tables

**Figure:** PRANCHA DE FIGURAS PARA AVALIAÇÃO DA FALA – BLOCO 1

**Fonte:** Marchesan^(22)^

**Nota:** Prancha atualizada pelos autores, com imagens geradas por inteligência Artificial (IA), a partir da versão original

**Figure:** PRANCHA DE FIGURAS PARA AVALIAÇÃO DA FALA – BLOCO 2

**Fonte:** Marchesan^(22)^

**Nota:** Prancha atualizada pelos autores, com imagens geradas por inteligência Artificial (IA), a partir da versão original.

## Apêndice A Protocolo de Exame Miofuncional Orofacial - MBGR reduzido (MBGR-r)^^ ***Marchesan IQ, Berretin-Felix G, Genaro KF, Rehder MI***

Nome:_______ N°:_______ Data do Exame: _______ /_______ /_______ Idade: _______ anos e meses DN: _______ /_______ /_______ Peso corporal: _______ kg Altura corporal: _______ m IMC: _______ (peso[kg]/estatura[m]^2^)

1. MEDIDAS DA FACE, DA OCLUSÃO E DOS MOVIMENTOS MANDIBULARES

Face, oclusão e movimentos mandibulares

**Table:**

		**Medida** (mm)
	Terço médio da face *(glabela^1^ a subnasal ^2^)*	
	Terço inferior da face *(subnasal ^2^ a gnatio ^5^)*	
	Lábio superior - LS *(subnasal ^2^ ao ponto mais inferior do lábio superior ^3^)*	
	Lábio inferior - LI *(do ponto mais superior do lábio inferior ^4^ ao gnatio ^5^)*	
	Trespasse vertical - TV *(dentes em oclusão, marcar no vestibular dos incisivos inferiores a face incisal dos incisivos superiores e medir a distância dessa marcação até a face incisal dos incisivos inferiores; na* *mordida aberta, medir a distância entre as faces incisais dos dentes incisivos superiores e inferiores no plano vertical e o resultado obtido será negativo)*	
	Trespasse horizontal - TH *(medir a distância entre as faces incisais dos incisivos superiores e inferiores no plano horizontal)*	
	Lateralidade mandibular ^6^ direita *(marcar a linha média dentária da arcada superior na arcada inferior,* *levar a mandíbula para a direita e manter, marcar novamente a linha média dentária da arcada superior na arcada inferior, voltar à posição de repouso e medir a distância entre as duas marcações)*	
	Lateralidade mandibular ^6^ esquerda *(marcar a linha média dentária da arcada superior na arcada* *inferior, levar a mandíbula para a esquerda e manter, marcar novamente a linha média dentária da arcada superior na arcada inferior, voltar à posição de repouso e medir a distância entre as duas marcações)*	
	Distância interincisal máxima ativa - DIMA *(do incisivo central ou lateral superior ao inferior com a máxima abertura da boca)*	

**2. EXAME EXTRAORAL [ ]** Soma dos pontos da face e lábios *(melhor resultado = 0 e pior = 14* )

**Face [ ]** (*melhor resultado = 0 e pior = 3*) *(na proporção facial, responder de acordo com medidas dos terços da face)*

**Table:**

Proporção facial:	(0) terço médio e inferior semelhantes	(1) terço inferior menor	(2) terço inferior maior
Padrão facial:	(0) padrão I (reto)	(1) padrão II (convexo)	(1) padrão III (côncavo)

Observação:_______________________________________________________________________________________________________

**Lábios [ ]** (*melhor resultado = 0 e pior = 11) (na proporção, responder de acordo com medidas de LS e LI)*

**Table:**

Postura habitual:		(0) fechados		(1) fechados com tensão		(2) ora abertos ora fechados	
		(2) entreabertos		(2) fechados em contato dentário		(3) abertos	
Forma do inferior:		(0) normal		(1) com eversão leve		(2) com eversão acentuada	
Exposição dos dentes incisivos superiores durante o repouso:			(0) cobre ⅔ dos incisivos		(1) cobre mais que ⅔		(2) cobre menos que ⅔
Proporção LS/LI:	(0) LS ½ da medida do LI				(1) LS > ½ da medida do LI		(2) LS < ½ da medida do LI
Mucosa externa:	(0) normal			(1) com saliva	(1) ressecada		(2) ferida

Observação:_______________________________________________________________________________________________________

**3. EXAME INTRAORAL [ ]** Soma dos pontos de lábios, língua, palato, tonsilas, dentes e oclusão

(m*elhor resultado = 0 e pior = 36*)

**Lábios [ ]** (*melhor resultado = 0 e pior = 2*)

**Table:**

Mucosa interna:	(0) adequada	(1) alterada (descrever):_________
Frênulo superior:	(0) adequada	(1) alterada (descrever):_________

Observação:_______________________________________________________________________________________________________

**Língua [ ]** *(melhor resultado = 0 e pior = 16) (mais de uma opção pode ser marcada para alguns aspectos e todas devem ser somadas para obtenção do escore)*

**Table:**

**Postura habitual:** ❒ não visível (1) no assoalho		(1) ponta baixa e dorso alto (1) interdental
**Largura:** (0) adequada (1) reduzida		(2) aumentada
**_Mucosa:_** (0) normal (1) geográfica (1) marcada por dentes (região):_____________________		(1) fissurada (2) ferida (região): _________________ (1) marcada por aparelho (região): _________________
**Frênulo:**	Fixação: no assoalho: (0) visível a partir das carúnculas na língua: (0) no terço médio	(1) visível a partir da crista alveolar inferior (1) entre o terço médio e o ápice (2) no ápice
	Forma do ápice ao elevar a língua: (0) arredondada	(1) quadrada ou retangular (1) ligeira fenda no ápice (2) formato de coração (3) não se eleva
	Espessura: (0) delgado	(0) submucoso (1) espesso

Observação:_______________________________________________________________________________________________________

**Palato [ ]** (*melhor resultado = 0 e pior = 8)*

**Table:**

Duro:	Profundidade:	(0) adequada	(1) reduzida (baixo)	(2) aumentada (alto)
Largura:		(0) adequada	(1) aumentada (larga)	(2) reduzida (estreitada)
Véu palatino: Simetria: Extensão:		(0) presente (0) adequada	(1) ausente (descrever):_____________ (1) longa (2) curta	
Úvula:	(0) adequada	(1) alterada (descrever):		

Observação:_______________________________________________________________________________________________________

**Tonsilas palatinas [ ]** (*melhor resultado = 0 e pior = 2)*

**Table:**

**Presença:** ❒ presentes	❒ removidas	❒ não visíveis
**Tamanho:** (0) adequado	(1) hipertrofia D	(1) hipertrofia E

Observação:_______________________________________________________________________________________________________

**Dentes [ ]** (*melhor resultado = 0 e pior = 1*)

**Table:**

Dentição:	decídua	dentadura mista ρ permanente	
Falha dentária:	(0) ausente	(1) presente (elementos):	

Observação:_______________________________________________________________________________________________________

**Oclusão [ ]** (*melhor resultado = 0 e pior = 7)*

**Table:**

Classificação de Angle:	Lado D: (0) Classe I	(1) Classe II div. 1ª (1) Classe II div. 2ª (1) Classe III
	Lado E: (0) Classe I	(1) Classe II div. 1ª (1) Classe II div. 2ª (1) Classe III
Relação horizontal:	(0) adequada [*1 a 3 mm*] (1) mordida de topo [*0 mm*]	(1) sobressaliência excessiva [*> 3 mm*] (1) mordida cruzada anterior [*< 0 mm*]
Relação vertical:	(0) adequada [*1 a 3 mm*] (1) mordida de topo [*0 mm*]	(1) sobremordida excessiva [*> 3 mm*] (1) mordida aberta posterior D (1) mordida aberta anterior *[< 0 mm*] (1) mordida aberta posterior E
Relação transversal:	(0) adequada	(1) mordida cruzada posterior D (1) mordida cruzada posterior E

Observação:_______________________________________________________________________________________________________

**4. MOBILIDADE [ ]** Somar os pontos de lábios, língua, véu palatino e mandíbula ( *melhor resultado = 0 e pior = 28* )

**Lábios [ ]** (*melhor resultado = 0 e pior = 9*) *(manter os dentes ocluídos*)*

**Table:**

	Adequada	Pequena alteração	Grande alteração	Não realiza
Protração de lábios fechados *:	(0)	(1)	(2)	(3)
Retração de lábios fechados*:	(0)	(1)	(2)	(3)
Protração de lábios fechados e alternância para direita e esquerda *:	(0)	(1)	(2)	(3)

Observação:_______________________________________________________________________________________________________

**Língua [ ]** (*melhor resultado = 0 e pior = 9*)

**Table:**

	Adequada	Pequena alteração	Grande alteração	Não realiza
Toque do ápice sequencialmente nas comissuras D/E e nos lábios S/I:	(0)	(1)	(2)	(3)
Toque do ápice na papila incisiva:	(0)	(1)	(2)	(3)
Sucção da língua no palato:	(0)	(1)	(2)	(3)

Observação:_______________________________________________________________________________________________________

**Véu palatino [ ]** (*melhor resultado = 0 e pior = 4*)

**Table:**

	Adequada	Reduzida	Não realiza
Emissão da vogal “a” repetidamente:	(0) Direita (0) Esquerda	(1) Direita (1) Esquerda	(2) Direita (2) Esquerda

Observação:_______________________________________________________________________________________________________

**Mandíbula [ ]** (*melhor resultado = 0 e pior = 6*)

**Table:**

	Adequada	Reduzida	Aumentada	Não realiza
Abertura da boca: *(DIMA + TV)*	(0)	(1)	(1)	(2)
	*VALORES ESPERADOS: criança = 35 a 50 mm / adulto = 40 a 55 mm*			
Lateralidade: direita: esquerda:	(0) (0)	(1) (1)	(1) (1)	(2) (2)
	*VALORES ESPERADOS: criança (6 a 12 anos) = 6 a 10 mm / adulto = 8 a 12 mm*			

Observação:_______________________________________________________________________________________________________

**5. TÔNUS [ ]** *(melhor resultado = 0 e pior = 6) (realizar observação visual e palpação)*

**Table:**

	Normal	Diminuído	Aumentado
Lábios:	(0) Superior (0) Inferior	(1) Superior (1) Inferior	(1) Superior (1) Inferior
Bochechas:	(0) Direita (0) Esquerda	(1) Direita (1) Esquerda	(1) Direita (1) Esquerda
Mento:	(0)	(1)	(1)
Língua:	(0)	(1)	(1)

Observação:_______________________________________________________________________________________________________

**6. FUNÇÕES OROFACIAS [ ]** Somar os pontos da respiração, mastigação, deglutição e fala

(melhor resultado = 0 e pior = 53)

**Respiração [ ]** *(melhor resultado = 0 e pior = 6)*

Se alterada, a origem é [ ] funcional [ ] estrutural [ ] outra:

**Table:**

**Modo:** (0) nasal (1) oronasal	(2) oral		
**Possibilidade de uso nasal:**	(0) 2 minutos ou mais	(1) entre 1 e 2 minutos	(2) menos que 1 minuto
**Fluxo nasal:** (0) adequado	(1) reduzido unilateral	(2) reduzido bilateral	

Observação:_______________________________________________________________________________________________________

**Mastigação Habitual [ ]** (*melhor resultado = 0 e pior = 8*) *(quando possível, utilizar sempre o mesmo alimento)*

Se alterada, a origem é [ ] funcional [ ] estrutural [ ] DTM [ ] outra:______________

**Table:**

**Incisão:** (0) anterior (1) lateral		(1) outra:
**Trituração:** (0) dentes posteriores (1) dentes anteriores		(1) com a língua
**Eficiência:** (0) adequada (1) alterada		
**Padrão mastigatório:**	(0) bilateral alternado *(50% - 65%)*	(0) unilateral alternado *(50% - 65%)* (0) unilateral preferencial *(66% - 75%)*
	(1) bilateral simultâneo (> 65%) (2) unilateral crônico (> 75%)	
**Fechamento labial:** (0) sistemático (1) assistemático		(2) ausente
**Ritmo:** (0) adequado (1) lento		(1) rápido

Observação:_______________________________________________________________________________________________________

**Deglutição Habitual de Sólido e Líquido***(água)***[ ]** (*melhor resultado = 0 e pior = 15*)

Se alterada, a origem é [ ] funcional [ ] estrutural [ ] outra:_________________

**Table:**

Postura da língua:	❒ não observável	(0) atrás dos dentes	(1) contra os dentes	(2) entre os dentes
Contenção do alimento:	(0) adequada	(1) inadequada sólido	(1) inadequada líquido	(2) inadequada sólido e líquido
Contração do orbicular:	(0) ausente	(1) acentuada sólido	(1) acentuada líquido	(2) acentuada sólido e líquido
Contração do mentual:	(0) ausente	(1) acentuada sólido	(1) acentuada líquido	(2) acentuada sólido e líquido
Movimento da cabeça:	(0) ausente	(1) presente sólido	(1) presente líquido	(2) presente sólido e líquido
Volume de líquido:	(0) satisfatório	(1) diminuído	(2) aumentado	
Ritmo de ingestão do líquido:	(0) sequencial	(1) gole por gole		
Ruído:	(0) ausente	(1) presente sólido	(1) presente líquido	(2) presente sólido e líquido

Observação:_______________________________________________________________________________________________________

**Fala [ ]** Somar os pontos da produção dos sons da fala e dos aspectos gerais *(melhor resultado = 0 e pior = 24)*

Se alterada, a origem é [ ] funcional [ ] estrutural [ ] fonológica [ ] outra:________________

**Produção dos sons da fala [ ]** *(melhor resultado = 0 e pior = 8) (avaliar por meio da fala espontânea, automática e nomeação de figuras)*

**Table:**

	Ausente	Presente Presente assistemática sistemática		Descrever
Omissão:	(0)	(1)	(2)	______________________________
Substituição:	(0)	(1)	(2)	______________________________
Distorção:	(0)	(1)	(2)	______________________________
Interdentalização	(0)	(1)	(2)	______________________________

Observação:_______________________________________________________________________________________________________

**Aspectos gerais [ ]** *(melhor resultado = 0 e pior = 16) (mais de uma opção pode ser marcada para alguns aspectos e todas devem ser somadas para obtenção do escore)*

**Table:**

**Saliva:** (0) deglutida (1) acumulada na comissura direita e/ou esquerda (1) acumulada no lábio inferior (2) espirra
**Abertura da boca:** (0) adequada (1) reduzida (1) aumentada
**Posição da língua:** (0) adequada (1) abaixada (1) anteriorizada (1) posteriorizada
**Movimento da mandíbula:** (0) trajetória adequada (1) desvio à direita (1) desvio à esquerda (1) anteriorização
**Articulação:** (0) precisa (1) imprecisão assistemática (2) imprecisão sistemática
**Velocidade:** (0) adequada (1) aumentada (1) reduzida
**Coordenação pneumofonoarticulatória:** (0) adequada (1) alterada:____________________
**Ressonância:** (0) equilibrada (1) uso reduzido nasal (1) uso excessivo nasal: ❒ leve ❒ moderada ❒ grave (1) uso excessivo laríngeo (1) uso excessivo faríngeo: ❒ leve ❒ moderada ❒ grave

Observação:_______________________________________________________________________________________________________

Dados coletados de exames:

Exames solicitados (justificativa):

Diagnóstico fonoaudiológico:

**Prognóstico:** ❒ favorável ❒ limitado ❒ desfavorável

Plano terapêutico:

Encaminhamento para outros profissionais (justificativa):

RESUMO DO EXAME MIOFUNCIONAL OROFACIAL - MBGR REDUZIDO (MBGR-r) ***Marchesan IQ, Berretin-Felix G, Genaro KF, Rehder MI***

**Table:**

**EXAME EXTRAORAL (melhor resultado = 0 e pior = 14)**	**[ ]**
Face (melhor resultado = 0 e pior = 3)	[ ]
Lábios (melhor resultado = 0 e pior = 11)	[ ]
**EXAME INTRAORAL (melhor resultado = 0 e pior = 36)**	**[ ]**
Lábios (melhor resultado = 0 e pior = 2)	[ ]
Língua (melhor resultado = 0 e pior = 16)	[ ]
Palato (melhor resultado = 0 e pior = 8)	[ ]
Tonsilas palatinas (melhor resultado = 0 e pior = 2)	[ ]
Dentes (melhor resultado = 0 e pior =1)	[ ]
Oclusão (melhor resultado = 0 e pior = 7)	[ ]
**MOBILIDADE (melhor resultado = 0 e pior = 28)**	**[ ]**
Lábios (melhor resultado = 0 e pior = 9)	[ ]
Língua (melhor resultado = 0 e pior = 9)	[ ]
Véu palatino (melhor resultado = 0 e pior = 4)	[ ]
Mandíbula (melhor resultado = 0 e pior = 6)	[ ]
**TÔNUS (melhor resultado = 0 e pior = 6)**	**[ ]**
Lábios (superior+ inferior) (melhor resultado = 0 e pior = 2)	[ ]
Bochechas (direita+ esquerda) (melhor resultado = 0 e pior = 2)	
Mento (melhor resultado = 0 e pior = 1)	[ ]
Língua (melhor resultado = 0 e pior = 1)	[ ]
**FUNÇÕES OROFACIAIS (melhor resultado = 0 e pior = 53)**	**[ ]**
Respiração (melhor resultado = 0 e pior = 6)	[ ]
Mastigação (melhor resultado = 0 e pior = 8)	[ ]
Deglutição (melhor resultado = 0 e pior = 15)	[ ]
Fala (melhor resultado = 0 e pior = 24)	[ ]
**PONTUAÇÃO TOTAL** (0 – 137 pontos)	

Fonoaudiólogo:_____________________________________________________________________

CRFª:_____________________________________________________________________

SUGESTÃO DE ROTEIRO PARA REGISTRO DE IMAGENS – MBGR REDUZIDO (MBGR-r)

Imagens estáticas

**Table:**

**Face:*** ❒ frente		❒ terço inferior de frente	❒ perfil direito em repouso
**Dentes:** ❒ arcada superior		❒ arcada inferior	
**Oclusão:** ❒ anterior		❒ lado direito	❒ lado esquerdo
**Língua:**	❒ em repouso no assoalho da boca ❒ protraída		
	❒ frênulo (língua elevada dentro da cavidade oral sem tocar o palato)		

*Com correção da postura da cabeça

Imagens Dinâmicas

**Table:**

Mobilidade:*	❒ lábios	❒ língua	❒ mandíbula ❒ véu palatino
Mastigação:	❒ habitual	❒ perguntas	
Deglutição Habitual:	❒ sólido	❒ líquido	
Fala:	❒ espontânea	❒ automática	❒ nomeação de figuras

*Solicitar três repetições de cada movimento

**Table:** QUADRO PARA ANOTAÇÃO DA AVALIAÇÃO DA FALA

Figura	Alteração presente	Figura	Alteração presente
Relógio		Barata	
Lápis		Morango	
Gato		Girafa	
Dado		Porta	
Passarinho		Barco	
Sofá		Garfo	
Tesoura		Prato	
Casa		Trem	
Bicicleta		Dragão	
Estrela		Livro	
Caminhão		Placa	
Olho		Flecha	
Chave		Blusa	
Avião		Flauta	
Borboleta		Sino	
Cachorro		Osso	
Telefone		Zebra	
Flor		Asa azul	
Presente		Guarda-chuva	
Jacaré		Chapéu	
Martelo		Janela	
Cruz		Joaninha	
Grama		Frango	
Coruja		Coroa	
Atleta		Globo	

**Fonte:** Quadro atualizado pelos autores, a partir da versão original do Protocolo de avaliação do frênulo da língua^(22)^
